# Evaluation of the Removal of Selected Phthalic Acid Esters (PAEs) in Municipal Wastewater Treatment Plants Supported by Constructed Wetlands

**DOI:** 10.3390/molecules26226966

**Published:** 2021-11-18

**Authors:** Daniel Wolecki, Barbara Trella, Fei Qi, Piotr Stepnowski, Jolanta Kumirska

**Affiliations:** 1 Department of Environmental Analysis, Faculty of Chemistry, University of Gdansk, Wita Stwosza 63, 80-308 Gdansk, Poland; basiatrella95@gmail.com (B.T.); piotr.stepnowski@ug.edu.pl (P.S.); 2 Beijing Key Lab for Source Control Technology of Water Pollution, College of Environmental Science and Engineering, Beijing Forestry University, Beijing 100083, China; qifei@bjfu.edu.cn

**Keywords:** phthalic acid esters, constructed wetlands, municipal wastewater, plant

## Abstract

Phthalic acid esters (PAEs) have a negative impact on living organisms in the environment, therefore, are among the group of Endocrine Disrupting Compounds (ECDs). Unfortunately, conventional methods used in municipal wastewater treatment plants (MWWTPs) are not designed to eliminate PAEs. For this reason, the development of cheap and simple but very effective techniques for the removal of such residues from wastewater is crucial. The main aim of this study was the evaluation of the removal of six selected PAEs: diethyl phthalate (DEP), di-*n*-octyl phthalate (DOP), di-*n*-butyl phthalate (DBP), benzyl butyl phthalate (BBP), bis(2-ethylhexyl) phthalate (DEHP) and dimethyl phthalate (DMP), in real MWWTPs supported by constructed wetlands (MWWTP–CW system). For the first time, the possibility of using three new plants for this purpose, *Cyperus papyrus* (papyrus), *Lysimachia nemorum* (yellow pimpernel) and *Euonymus europaeus* (European spindle), has been presented. For determining the target PAEs in wastewater samples, a method of SPE (Solid-Phase Extraction)–GC–MS(SIM) was developed and validated, and for plant materials, a method of UAE (Ultrasound-Assisted Extraction)–SPE–GC–MS(SIM) was proposed. The obtained data showed that the application of the MWWTP–CW system allows a significant increase in the removal of DEP, DBP, BBP and DEHP from the wastewater stream. *Euonymus europaeus* was the most effective among the tested plant species for the uptake of analytes (8938 ng × g^−1^ dry weight), thus, this plant was found to be optimal for supporting conventional MWWTPs.

## 1. Introduction

Phthalic acid esters (PAEs), usually called phthalate esters or just phthalates, were introduced into the industrial sector in the 1930s as plasticizers to give plastics their intended shape [1]. Since then, PAEs have been used in packaging materials, textiles, medical equipment and the production of electronics [2]. In some synthetics, such as polyvinyl chloride (PVC), phthalates account for 60% of the weight of the produced material [3]. By 2011, world production of plasticizers amounted to 8 million tons, and according to scientific reports, it is growing steadily [4]. Phthalic acid esters in living organisms can induce an effect like estrogens; therefore, PAEs are among the group of Endocrine Disrupting Compounds (ECDs). Data from in silico experiments have shown that some PAEs cause harmful effects on mammalian and non-mammalian organisms (e.g., [5]). For example, dibutyl phthalate (DBP) and benzyl butyl phthalate (BBP) and their metabolites were shown to be indicative of hemolysis and eryptosis in human erythrocytes [6]. For this reason, in many countries around the world, appropriate regulations have been introduced regarding the use and marketing of phthalate-containing products. For example, bis(2-ethylhexyl) phthalate (DEHP), dibutyl phthalate (DBP), benzyl butyl phthalate (BBP) and diisobutyl phthalate (DIBP) are included in Commission Regulation (EU) 2018/2005, amending Annex XVII to Regulation (EC) No 1907/2006 of the European Parliament and the Council concerning the Registration, Evaluation, Authorization and Restriction of Chemicals (REACH) [7]. However, the constant release of phthalates causes the accumulation of PAEs in different environmental niches [8]. For example, the presence of PAEs has already been confirmed in sediments and marine organisms of the Baltic Sea, which is a significant environmental threat [9].

Conventional methods used in wastewater treatment plants (WWTPs) for wastewater treatment are not designed to eliminate PAEs [10] and under typical conditions, only 18% of PAEs can be removed by WWTPs. For this reason, wastewater leaving WWTPs is one of the main sources of PAEs in the environment [4,11,12]. Since conventional activated sludge (AS)-based WWTPs are inefficient in the removal of PAEs, the implementation of additional wastewater cleaning processes is necessary. Thus, the development of efficient and cheap methods of wastewater treatment regarding the removal of PAEs is an important task for the protection of the environment and environmental engineering.

**Figure 1:**
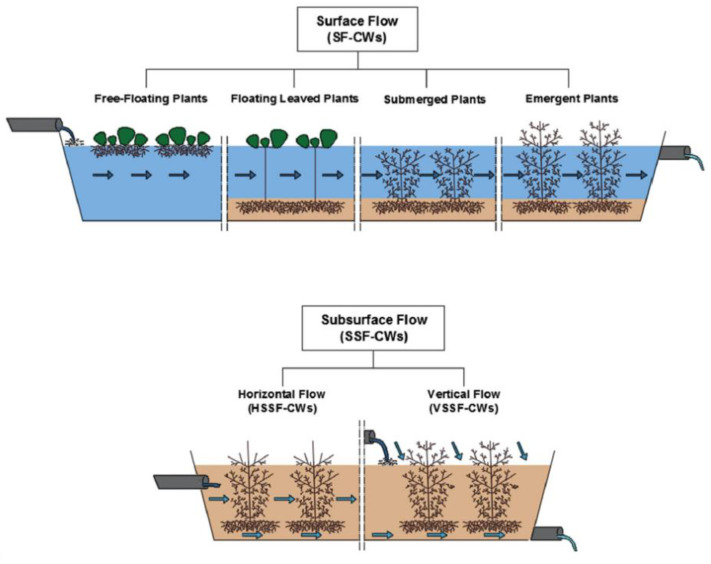
CWs configurations, according to hydrology (surface or subsurface flow), plant growth forms for surface flow (free-floating, floating leaved, submerged or emergent) and flow path for subsurface flow (horizontal or vertical). Reprinted from Environmental Pollution, Vol 227, Ana M. Gorito, Ana R. Ribeiro, C.M.R. Almeida, Adrian M.T. Silva, A review on the application of constructed wetlands for the removal of priority substances and contaminants of emerging concern listed in recently launched EU legislation, Pages No. 428–443, Copyright (2017) [10], with permission from Elsevier. (License Number 5166980675287).

Constructed wetlands (CWs) are a green chemistry approach, where plants capable of growing in highly hydrated conditions are used to remove contaminants [13,14]. CWs are systems in which water, soil, plants and microorganisms interact to greatly benefit the elimination of pollutants. In most cases, CWs in WWTPs are a separate sector of the wastewater treatment process, implemented after biological treatment. They include the following configurations: surface flow constructed wetlands (SFCWs), horizontal surface flow constructed wetlands (HSSFCWs) and vertical subsurface flow constructed wetlands (VSSFCWs) [10] (Figure 1).

CWs could be also implemented within the second stage of wastewater treatment (biological). Contact between the plants and wastewater (mixed with AS) can occur only in the rhyzophytic zone.

In comparison to classic pollutants (such as biogens or organic substances), CWs are the perfect complement to wastewater treatment technology [15]. The removal mechanism of contaminants in CWs is complicated and consists of physical, chemical and biological processes among the plants, substrates and microorganisms. It can also be affected by the type of CW, the substrate type and the plants used [10]. The literature data [16,17,18,19,20,21,22,23,24,25,26,27,28] describing the use of CWs in WWTPs for the effective neutralization/sorption/degradation of phthalates are very limited (Table S1, Supplementary Material). Most investigations are performed using a laboratory system [16,17,18,19,20,21] or a pilot-scale system [22,23,24,25,28]. A full-scale constructed wetland experiment is only presented in a few papers [26,27]. Moreover, the uptake of PAEs by plants in CWs is seldom investigated. Such data are only presented for a laboratory system and such plants as *Phragmites australis* by Li et al. [16], and *Brassica juncea* and *Helianthus annuus* by Zavoda et al. [21]. The uptake of PAEs by *Typha* plants in working full-scale CW systems was investigated only recently by Diepenheim et al. [27]. One of the main reasons for the sparse research is the limited number of analytical methods developed for the determination of PAEs in plant materials. The literature data available on this topic [2,29,30,31,32,33] are presented in Table S2, and in most cases, the papers describe the phytoremediation of PAEs by vegetables planted in contaminated soils.

Taking into account the information presented above, the main aim of this study was to evaluate the removal of PAEs in a working municipal wastewater treatment plant (MWWTP) supported by CWs by the simultaneous determination of PAEs in raw and treated sewage, in addition to the determination of the uptake of the target compounds by plants in CWs. Contrary to the working full-scale CW system investigated by Diepenheim et al. [27], where an operational horizontal subsurface flow CW received effluent from an adjacent WWTP, in this study CWs were introduced in the stage of biological wastewater treatment. Moreover, for the first time, the possibility of using three new plants for this purpose: *Cyperus papyrus* (papyrus), *Lysimachia nemorum* (yellow pimpernel) and *Euonymus europaeus* (European spindle), has been presented. However, because appropriate analytical methods for the simultaneous determination of the six target phthalates (Table S3, Supplementary Material) in wastewater and CW plants were not available, the main objectives of this work were as follows: (1) the modification of an analytical method for the determination of PAEs in raw and treated sewage water; (2) the modification of an analytical method for the determination of selected PAEs in plant materials from CWs; (3) the determination of selected phthalates in raw and treated sewage samples from an MWWTP supported by CWs; (4) an assessment of the uptake of PAEs by plants used in CWs; (5) an evaluation of the possibility of using hydroponic cultivations for the effective removal of PAEs in MWWTPs.

According to information included in the literature [2,29,30,31,32,33], the most frequently used technique for the isolation of PAEs from plant materials is Ultrasound-Assisted Extraction (UAE) [2,27,31,32,33] (Tables S1 and S2). In this study, we decided to verify the usefulness of this technique for the isolation of the target compounds from the plants: *Cyperus papyrus* (papyrus), *Lysimachia nemorum* (yellow pimpernel) and *Euonymus europaeus* (European spindle). Such investigations were not performed in any of the published papers.

## 2. Results and Discussion

### 2.1. Evaluation of the Analytical Method for Determining the Target Compounds in Sewage Samples

**Figure 2:**
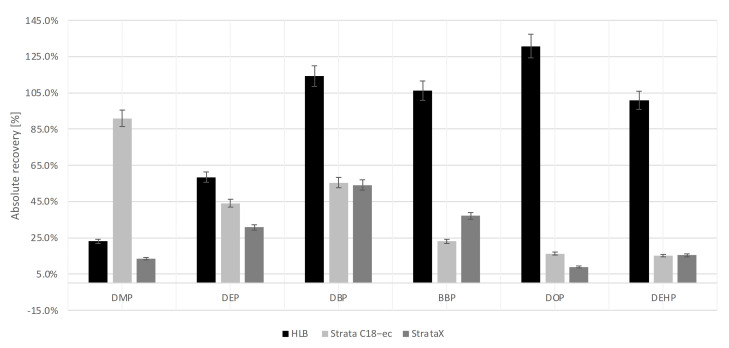
Absolute recovery (AR ± SD, %) of six target PAEs using different types of SPE cartridges (Oasis HLB, Strata C18-ec and Strata X; n = 3).

Three different SPE cartridges, Oasis HLB, Strata C18-ec and Strata X, were tested in order to find the optimal conditions for the extraction of the target compounds from wastewater samples (Section 3.4). The absolute recoveries (%) of the target PAEs extracted from distilled water spiked with phthalates to a concentration of each analyte of 4 µg × L^−1^ and acidification to pH 3 are shown in Figure 2. All obtained extracts were analyzed using the GC–MS(SIM) method presented in Section 3.6.

Among the tested SPE cartridges, the most effective was the hydrophilic–lipophilic balance water-wettable sorbent, namely, Oasis HLB (Figure 2). The AR values of DBP, BBP, DOP and DEHP were 114% ± 1%, 106% ± 6%, 131% ± 6% and 101% ± 6%, respectively. For more polar phthalates such as DMP and DEP, the AR values were lower (Figure 2). Measurement precision, expressed by the standard deviation (SD), was satisfactory and did not exceed 6%. Surprisingly, the application of the Strata X cartridge with a solid-phase structure similar to Oasis HLB was not satisfactory enough for the extraction of PAEs. On the other hand, the SD values in this case were the lowest among all those recorded for the tested cartridges. Summarizing, the Oasis HLB cartridge with the extraction sequence described in Section 3.4 was chosen as optimal for the extraction of the target PAEs from water samples.

In order to fully optimize the analytical method, raw and treated sewage samples containing trace levels of PAEs were spiked with a known amount of the target phthalates (250 ng × L^−1^, 500 ng × L^−1^ and 1000 ng × L^−1^) and subjected to extraction. The analysis of non-spiked wastewater samples was also performed. In both matrix types, untreated and treated sewage samples, the absolute recovery data were evaluated; the results are presented in Table 1.

The literature data concerning studies of the usefulness of hydroponically cultivated plants for removing PAEs from the sewage stream do not present the absolute recoveries of analytes in the development of the applied analytical methods, yet sometimes recoveries calculated based on surrogate solutions are presented. (Table S1, Supplementary Material). Some show only the concentrations of PAEs in the sewage/water samples without details describing the applied analytical protocols, and sometimes only the theoretical concentration in the water phase was considered in such studies [2]. The recoveries of DMP, DEP, DBP, BBP and DOP from water samples using Oasis HLB cartridges during SPE–GC–FID analyses reported by Xiaoyan et al. (2015) ranged from 74% to 108% [20]. Reyes-Contreras and co-workers (2011) used a previously developed method for determining pharmaceuticals in CWs in order to determine phthalates in wastewaters and established that the recoveries of all analytes were always above 90% [22]. Similar results were obtained by Masi et al. (2004), who applied a liquid/liquid extraction procedure with GC–MS measurements; the established recoveries ranged from 60% to 97% [24]. According to Zhao et al. (2004), the recovery of DBP from water samples based on a C_18_ SPE procedure coupled with HPLC was 141.2% [25]. Diepenheim et al. [27] developed an SPE–GC–MS method for the simultaneous determination of 15 PAEs in wastewater, including the phthalates investigated in this study. They used 47 mm HLB Oasis disks for their extraction. PAEs were eluted using 2 × 3 mL of hexanes and 3 mL of ethyl acetate. Next, the samples were concentrated, exchanged to hexanes and spiked with the internal standard [27]. The average recoveries for the laboratory control samples (500 to 1500 ng of phthalates) were 79% ± 38%. The matrix spike average recoveries were 100% (a range from 32% to 137%) for water [27].

Jiang and co-workers (2018) [34] developed an SPE–LC–MS/MS method for the simultaneous determination of primary and secondary phthalate monoesters, which was applied to determine these compounds in the Taihu Lake surface water. Unfortunately, only the recoveries for primary monoesters of phthalic acid were presented (a range from 76.8 to 86.2%) [34]. Ballesteros and co-workers (2006) [35] determined phthalate esters, alkylphenols, bisphenol A and their chlorinated derivatives in wastewater samples using an SPE–GC–MS method. The recoveries of PAEs based on a surrogate solution (bisphenol F) (DMP 96.0–105.0%, DEP 95.6–98.0%, DBP 96.0–103.4%) [35] were similar to those presented in Table 1. However, because the recoveries were calculated based on the surrogate solution, a direct comparison with the absolute recoveries presented in this paper is not possible. Adewuyi (2012) determined DMP, DEP, DPhP, DBP and DEHP in sewage and water samples using liquid–liquid extraction (dichloromethane as the extraction medium), the purification of the extracts on a column of silica gel with hexane as the mobile solvent and an analysis of the cleaned extracts by HPLC [36]. The recoveries of analytes, calculated based on an internal standard (IS), ranged from 57.48% (DPhP) to 96.95% (DEHP) [36]. In summary, it can be concluded that the recoveries of analytes presented in this study were similar to those in the literature [20,22,24,25,34,35,36]. However, in this study, such data are shown as absolute recoveries, whereas in the cited literature they are shown mostly as relative recoveries without the presentation of absolute recoveries.

### 2.2. Evaluation of the Analytical Method for Determining the Target Compounds in Plant Materials

Knowledge of the impact of the plant species in CWs on the removal of phthalates from wastewater requires an assessment of the uptake of these compounds by plants. Based on the literature data (Table S1, Supplementary Material), the required methodology was evaluated and the concentrations of compounds in CW plant materials were established: DBP for *Phragmites australis* [16], DBP, DOP and BBP for *Brassica juncea* and *Helianthus annuus* plants [21] as well as for *Typha* (15 PAEs) [27]. Moreover, such studies were conducted mainly for laboratory systems, and only in one case for a working full-scale MWWTP–CW system [27] (details in Table S1 in Supplementary Material). Thus, the development of an analytical procedure for determining the six target PAEs in the plants selected in this study was necessary. For this reason, after the development of the GC–MS(SIM) method described in Section 3.6 for the final determination and evaluation of the extraction efficiency of the target compounds from sewage samples, our research focused on the optimization of the extraction of phthalates from plant materials. As mentioned in the Introduction, based on the Supplementary Material (Tables S1 and S2), the most frequently used technique for the isolation of PAEs from plant materials is UAE [2,27,31,32,33]. We decided to optimize the UAE conditions (the application of different extraction solvents, ethyl acetate (EtOAc), methanol (MeOH) and dichloromethane (DCM)), and to use the previously proposed SPE procedure for the purification of the obtained extracts.

The results of the optimization of the UAE procedure are presented in Table 2. The best absolute recoveries of the target PAEs (ranging from 32 ± 4% (DMP) to 130 ± 31% (DOP)) with the lowest standard deviation (SD) were obtained during the application of DCM as the extraction solvent. With the use of MEOH, lower AR values were observed for all PAEs, and for DEHP this value was unsatisfactory (14 ± 3%) (Table 2) (acceptable criteria for AR >30% [37]). During the application of EtOH as the extraction medium, better absolute recovery results were observed for DEP (60 ± 16% instead of 43 ± 8%) and DBP (102 ± 31% instead of 83 ± 6%); however, the SD values were much higher than those observed for extraction using DCM. Moreover, DOP was not isolated in these conditions.

In order to compare the recovery data obtained in this work with those presented by other research groups for PAEs extracted from plant materials, we collected the literature data assessing the uptake of these compounds by plants, respectively in Tables S1 and S2. Unfortunately, only four papers [16,27,29,33] presented recovery data. Li et al. [16] reported that the recovery of DBP from *Phragmites australis* based on a UAE–GC–MS method was 89%. Diepenheim et al. [27] established that the matrix spike average recoveries of analytes for plant samples were 72% (a range from 0% to 121%). Liao et al. [29] confirmed that the recovery percentage of DBP isolated from garden lettuce (*Lactuca sativa* L. var. *longifolia*) was 96.5%. Sun et al. [33] proved that the recoveries of DBP and DEHP from lettuce *Lactuca sativa*, strawberry *Fragaria × ananassa* and carrot *Daucus carota *Var.* Sativa,* calculated based on internal standards, ranged from 75 to 110%. Sablayrolles et al. (2005) developed and validated a method of extracting phthalates from sludge and vegetables [38]. The authors determined the same phthalates as in our study but the isolation of PAEs from plant materials was performed using the Soxhlet extraction method with *n*-hexane as the extraction matrix and the purification of the obtained extracts by SPE (Florisil). Relative recoveries (internal standard; DEHP-d_4_) ranged from 100 to 101% [38]. Holadová and Hajšlová (1995) [39] described the development of a method for determining the same phthalates as in our study, using homogenization with a hexane/acetone (2:1, *v/v*) mixture and GC–ECD. Based on deuterated standards (DEHP-d_4_ and DBP-d_4_), the relative recoveries of PAEs from lettuce samples ranged from 50 to 120%. Generally, our research presents absolute recovery data, not relative recoveries, so a direct comparison of the obtained results with those presented in other studies is problematic. Taking into account that the criterion acceptable for AR is above 30% [37], the proposed UAE–SPE procedure fully fulfills this requirement.

As mentioned, the chromatographic conditions of the GC–MS measurements of plant extracts are presented in Section 3.6.

### 2.3. Validation Parameters of the Proposed SPE–G–MS(SIM) and UAE–SPE–GC–MS(SIM) Methods for Determining Phthalates in Wastewater and Plants

The developed methods for determining target PAEs in wastewater and plants were validated using working calibration standard solutions and matrix-matched calibration solutions according to the guidelines of the International Vocabulary of Metrology [40] and procedures fully described in our previous papers [41,42] (Section 3.7). The determined validation parameters are presented in Table 3. The coefficient of determination (R^2^) ranged from 0.9941 to 0.9986 and the intermediate precision measurement from 0.2 to 9.2%. Accuracy, expressed by the mean recovery (MR), based on the determined and known concentrations of analytes, was between 80% and 114% for plants and between 80% and 120%, and 80% and 119% for raw and treated wastewater, respectively (Table 3). Matrix effects (ME) for plants ranged from −24 ± 4% for DMP to 3 ± 1% for DEHP. For wastewater samples, ME values were between −25 ± 6% and +50 ± 14% for untreated wastewater, and between −35 ± 7% and +34 ± 9% for treated wastewater (Table 3). A comparison of the obtained ME values with those presented by other authors was not possible because matrix effects were not presented in the cited papers (Tables S1 and S2). Fernández-González et al. (2017) determined the matrix effects for the HS–SPME–GC–MS determination of phthalates in sediment samples [43]. They proved that the ones for DMP, DEP, DBP, and BBP were negligible. However, ME values for DEHP and DOP were 40% and 60%, respectively. In our opinion, the matrix effects determined in this study, which did not exceed 50%, in combination with other validation parameters (Table 3) and ME data presented for environmental matrices [37,43], are satisfactory.

The method quantification limit and method detection limit values were almost the same or similar to those presented in other research [2,16,17,18,19,20,21,22,23,24,25,26,27,28,29,30,31,32,33].

**Table 3:** Selected validation parameters of the developed methods for determining target compounds in wastewater and plant samples from an MWWTP (analytical range from MQL to 2500 ng × g^−1^ for plant samples, and from MQL to 1000 ng × L^−1^ for wastewater samples, n = 3). Abbreviations: MR—mean recovery; ME—matrix effect; MQL—method quantification limit; MDL—method detection limit; UW—untreated wastewater; TW—treated wastewater.

Validation Parameters	Calibration Curves	R^2^	Intermediate Precision Measurement (RSD) %	MR [%]			ME [% ± SD]			MQL; MDL [ng × L^−1^ or ng × g^−1^ d.w.]		
Compounds				UW	TW	Plants	UW	TW	Plants	UW	TW	Plants
**DMP**	17988.73X + 683.92	0.9960	2.0–5.8	80–111	84–119	83–111	+17 ± 9	+34 ± 9	−24 ± 4	5; 2	6; 2	30; 10
**DEP**	45676.99X + 1352.61	0.9965	1.3–5.9	90–120	80–118	80–112	+45 ± 11	+8 ± 2	−17 ± 3	6; 2	7; 2	23; 8
**DBP**	117497.40X + 348.26	0.9956	2.3–9.2	85–114	90–111	90–113	−25 ± 6	−13 ± 4	−10 ± 1	5; 2	6; 2	12; 4
**BBP**	51801.06X − 711.14	0.9960	0.2–8.0	90–118	95–105	90–114	+40 ± 17	+14 ± 4	−6 ± 1	3; 1	4; 1	12; 4
**DOP**	110988.50X − 1483.44	0.9986	0.8–8.1	95–106	90–108	94–108	+50 ± 14	−35 ± 7	−10 ± 2	5; 2	7; 2	8; 3
**DEHP**	144296.80X − 6362.41	0.9941	1.8–5.9	80–104	85–115	90–103	+29 ± 8	−30 ± 5	3 ± 1	6; 2	4; 1	14; 5

### 2.4. Determination of Selected Phthalates in Wastewater and Plant Materials from an MWWTP

#### 2.4.1. Assessment of the Presence of Phthalates in Raw and Treated Wastewater

The method for determining target PAEs in raw and treated wastewater was described in Section 2.1 and 3.4. The identification of analytes was performed based on the retention time, quantitative ion and confirmation ions, described in Section 3.6. The mass spectra of the target compounds with the assignation of MS fragments are included in Figure S2 in Supplementary Material. The determined concentrations of the six target compounds in untreated and treated sewage collected from the studied full-scale MWWTP supported by CWs, characterized in Section 3.2, are presented in Table 4.

In this study, for the first time in Poland and this part of Europe, the concentrations of phthalates, which pose a danger to living organisms, in sewage derived from a full-scale MWWTP supported by CWs were investigated. In both raw and treated sewage samples, DMP was not found (concentration below the method detection limit (MDL)). Two phthalates, DEP and DBP, were found in the highest concentrations in raw sewage at 10,097 ± 202 ng × L^−1^ and 6196 ± 805 ng × L^−1^, respectively (Table 5). BBP, DOP and DEHP were determined at the concentrations 204 ± 2 ng × L^−1^, 221 ± 7 ng × L^−1^ and 136 ± 0 ng × L^−1^, respectively, in raw sewage. The concentrations of DEP and DBP, as well as BBP and DEHP, were lower in treated sewage in comparison to raw sewage, with the biggest differences observed for DEP and DBP (Table 4). Only the concentration of DOP in treated sewage was 20% higher than in raw sewage. The probable reason for this result could be the additional pollution of the wastewater by active and passive elements of the WWTP, made of plastic. In order to prove this result, a repetition analysis was performed, and again a higher DOP concentration in treated wastewater was observed. Moreover, we have performed an analysis of wastewater samples using a full scan GC–MS system with the registration of the total ion chromatogram (TIC). No co-elution or signal overlap was observed. The exemplary TICs recorded for raw (A) and treated (B) wastewater samples are presented in Supplementary Materials in Figure S3. A similar situation was observed by Gao et al. (2014) who determined the concentrations of phthalates in untreated and treated sewage, and also determined a higher concentration of DOP in treated sewage (mean concentration 9.22 ng × mL^−1^) than in untreated (mean concentration 8.08 ng × mL^−1^) [44]. The mean concentration of BBP in the effluent (8.3 ng × mL^−1^) was also higher than this one in the influent (3.66 ng × mL^−1^) [44].

Our results confirmed the presence of target PAEs in raw and treated sewage from WWTPs (Table 4). Similar data are presented in other research (Table S1). To the best of our knowledge, only two studies [26,27] reported the determination of phthalates in full-scale CWs (Table S1). CWs were found to have a negative effect on PAE concentrations in sewage in both summer and winter [26], whereas positive results were reported by Diepenheim et al. [27]. Some studies [22,23,24,25,28] describe pilot-scale CWs or those existing as stand-alone hydroponic crops [17,18,19,20,21] (Table S1).

Gani and Kazmi (2016) determined DEP, DBP, BBP and DEHP in sewage and their removal in a sequencing batch reactor (SBR), activated sludge process (ASP) and up-flow anaerobic sludge blanket (UASB) reactor [45]. They reported the concentrations of these compounds in raw wastewater at 5417 ± 4149 ng × L^−1^, 11,175 ± 9977 ng × L^−1^, 1968 ± 2280 ng × L^−1^ and 27,011 ± 14,341 ng × L^−1^, respectively [45]. In treated wastewater, DBP and DEHP concentrations were much higher than those reported in our research, 2188 ± 1847 ng × L^−1^ and 4253 ± 2521 ng × L^−1^, respectively.

#### 2.4.2. Assessment of the Uptake of Phthalates in Hydroponically Cultivated Plants

The determined concentrations of phthalates in three species of hydroponically cultivated plants (in ng × g^−1^ dry weight) and the elimination efficiency (EE) of phthalates calculated by Equation 2, described in Section 3.9, are presented in Table 5. Example chromatograms with marked SIM ions for the determined target compounds in real papyrus (*Cyperus papyrus*), yellow pimpernel (*Lysimachia nemorum*) and European spindle (*Euonymus europaeus*) samples are included in Figures S4–S6, respectively.

Among the six determined phthalic acid esters, three of them (DEP, DBP and DOP) were found in all the tested plant species (*Cyperus papyrus*, *Lysimachia nemorum*, *Euonymus europaeus*). Additionally, in *E. europaeus,* DOP was determined at the highest concentration of 6562 ± 1065 ng × g^−1^ d.w., as well as DEP and DMP, at 477 ± 83 ng × g^−1^ d.w. and 397 ± 12 ng × g^−1^ d.w., respectively (Table 5). *C. papyrus* plant material contained DEP, DBP, BBP and DOP, whereas DMP and DEHP were not found. In *Lysimachia nemorum* only BBP was not identified. DMP was determined at a concentration of 98 ± 2 ng × g^−1^ d.w., DBP at a concentration of 1697 ± 140 ng × g^−1^ d.w., DOP at 1343 ± 193 ng × g^−1^ d.w. and DEHP at 53 ± 22 ng × g^−1^ d.w. (Table 5).

Few studies [16,21,27] have directly summarized the uptake of phthalates by plants in CWs (Table S1). Li et al. (2020) investigated the possibility of the uptake of DBP by the *Phragmites australis* plant in laboratory CWs [16]. The target compound was detected in plant tissue in variable concentrations during spring, summer and autumn, with the highest concentration (0.468–4.000 µg/g d.w.) detected in autumn. Moreover, the authors concluded that DBP was preliminarily removed by biodegradation in this system and the uptake and substrate adsorption of *P. australis* were negligible for the removal of DBP [16]. Zavoda et al. [21] investigated the ability of dwarf sunflowers (*Helianthus annuus*) and two strains of mustard seed (*Brassica juncea*) to hydroponically treat water contaminated with the phthalates DBP, DOP and BBP. For individual phthalate treatment, the selectivity was DBP > BBP > DOP. The sunflowers had a better uptake rate and a higher concentration of contaminants in plant tissue than the mustard seed (over 100 ppm of phthalates during a 4-day study). Diepenheim et al. [27] studied the distribution of 15 phthalates in water, sediment and two dominant plant species (*Typha latifolia*, *Typha angustifolia*) in a fully operational CW (Table S1). In contrast to our study, an operational horizontal subsurface flow CW received effluent from an adjacent WWTP. Plant species were sampled by collecting the portions emerging above the surface of the water. The average dry weight of Σ15 phthalates was 1.23 ± 0.53 μg × g^−1^ for *Typha* shoots; DBP and DEHP were most often found. Significant concentrations of DBP, DEHP and other water-soluble phthalates in the shoots of *Typha* (for comparison: 0.088–2.02 μg × g^−1^ d.w. for DBP) indicated that plant uptake is a potentially important removal mechanism of phthalates in water exiting the CW [27].

In this study, the obtained elimination efficiency (EE) of the investigated phthalates from wastewater ranged from 56% for BBP to 98% for DEP (Table 5). In comparison, the literature EE data for DBP, presented in Table S1, are as follows: >89.7% [16], 62.08–84.17% [17], 87.2% [19], 99.99% [23] and 99.84% [25]. In our study, the elimination efficiency of DBP from wastewater was 94%, which confirms a similar or higher elimination efficiency than presented by other studies. Lower EE values for DEP than the value determined in this study (98%) were presented in [18] (48.2–61.5%: Exp. I 144 µg/L in sewage; 55.8–67.5: Exp. II 150 µg/L in sewage), in [20] (44–83%) and in [22] (in winter ~73%) (Table S1). Summarizing, the obtained results proved that the uptake of PAEs by plants in an MWWTP supported by CWs resulted in an increase in the effectiveness of the removal of such compounds from wastewater.

#### 2.4.3. Assessment of the Usefulness of Hydroponically Cultivated Plants for Removing Phthalates from the Sewage Stream

In accordance with the literature data, the most frequently hydroponically cultivated plants are *Typha sp*., *Phragmites sp*. and *T. aestivum* (Table S1) due to the considerable size of the rhizomes and roots. In this study, the three plants, *Cyperus papyrus* (papyrus), *Lysimachia nemorum* (yellow pimpernel) and *Euonymus europaeus* (European spindle), were evaluated for the first time for this purpose. The above-mentioned plants are dominant since they adapt very well to growth in MWWTPs and show the strongest development during the growing season. To assess the usefulness of these species for removing phthalates from the wastewater stream, the sum of the uptake masses of phthalates taken by the tested species was calculated, and the results are presented in Table 6.

Accordingly, the highest uptake of target compounds was observed for *Euonymus europaeus* (European spindle) (8938 ng × g^−1^ d.w.), followed by *Cyperus papyrus* (papyrus) (5737 ng × g^−1^ d.w.), and the lowest uptake for *Lysimachia nemorum* (yellow pimpernel) (3504 ng × g^−1^ d.w.). Summarizing, the *Euonymus europaeus* species (European spindle) is the best of the tested plants for the uptake of the target phthalates. The different sums of the uptake of PAEs are related to the morphological structures of the tested plants. *E. europaeus* and *C. papyrus* have a well-developed system of roots and rhizomes, thus, effectively delivering nutrients to the green parts of the plant. In addition, they show strong growth during the growing season, of up to 3 m. *L. nemorum* is an evergreen creeping perennial herbaceous plant, growing to approximately 40 cm, with a poorly developed rhizome system but an extensive root. The determination of PAEs in influent and effluent wastewater and in plant tissues allowed for the determination of which hydroponic cultivation system most significantly supported the wastewater treatment process (Table 4, Table 5 and Table 6).

## 3. Materials and Methods

### 3.1. Chemicals and Materials

EPA Method 8091A Phthalate Ester Mixtures containing the target compounds dimethyl phthalate (DMP), diethyl phthalate (DEP), di-*n*-butyl phthalate (DBP), benzyl butyl phthalate (BBP), di-*n*-octyl phthalate (DOP) and bis(2-ethylhexyl) phthalate (DEHP), (>99%) in hexane:acetone (8:2, *v/v*) at concentrations of 0.1 mg × L^−1^ were obtained from Restek (Bellefonte, PA, USA). High-purity analytical grade trace solvents methanol, dichloromethane and acetonitrile were supplied by POCH (Gliwice, Poland); ethyl acetate and acetone were purchased from Merck (Sigma Aldrich, Darmstadt, Germany). For the acidification of extracts, 37% hydrochloric acid (HCl) was provided by Chempur (Piekary Śląskie, Poland). Anhydrous magnesium sulfate was purchased from Eurochem BGD Sp. z o.o. (Tarnów, Poland). Solid-phase extraction (SPE) procedures were optimized using testing cartridges such as Oasis HLB (6 mL, 200 mg, Waters Corporation, Milford, MA USA), StrataX (3 mL, 200 mg, Phenomenex, Aschaffenburg, Germany) and Strata C18-ec (6 mL, 500 mg, Chromabond, Macherey-Nagel, Düren, Germany). Standard stock solutions of the target compounds (10 µg × mL^−1^) were prepared in acetone. All the stock solutions were stored at –20 °C. Working calibration standard solutions were prepared by diluting the standard stock solutions in the appropriate amounts of acetone, and they were stored in the dark at –20 °C.

The chemical structures and physicochemical properties of the target PAEs in this study are presented in Table S3 in the Supplementary Materials.

### 3.2. Characterization of the Studied Full-Scale MWWTP with CWs

The investigations were performed at the full-scale municipal wastewater treatment plant (MWWTP) in Sochaczew (Mazowieckie Voivodeship, central Poland), which combines the method of biological wastewater treatment with AS and CWs (Figure S2 in the Supplementary Materials). This MWWTP was fully described in our previous paper [41]. Briefly, it is designed for a 55,925-equivalent population with a maximum daily volume of sewage at 11,636 m^3^ × d^−1^. The wastewater collection from the area of Sochaczew city concerns the domestic inflow from approximately 37,000 residents. The MWWTP consists of the following elements: (1°) the mechanical part of wastewater treatment including a drainage station, mechanical wastewater treatment using gratings and aerated sandboxes with degreasers (grates with a throughput of 515 m^3^ × h^−1^, aerated sand traps with degreasers, aerated at 1.91 m^3^ × min^−1^); (2°) the biological part of wastewater treatment containing a flow reactor with AS with CWs and a secondary settler with recirculated sludge (flow reactor with AS with a throughput of 6000 m^3^ × d^−1^ and a secondary settling tank with an active capacity of 1142 m^3^); (3°) a dehydration and liming sludge station. CWs are introduced at the stage of biological wastewater treatment (2°); contact between the plants and wastewater (mixed with AS) occurs only in the rhyzophytic zone (Figure S2 in the Supplementary Materials). CW plants are placed in a greenhouse with a total area of 1835.6 m^2^, where the optimal air humidity and temperature (35–38 °C) are maintained for appropriate plant growth (Figure S2). The effluent from the MWWTP is discharged into the Utrata River. The average values of the main MWWTP technological parameters are presented in Table S4 in the Supplementary Materials.

### 3.3. Sampling Wastewater and Plant Materials from CWs

Both types of wastewaters (raw and treated) were collected in November 2017, in amber glass bottles (2.5 L), appropriately primed for the analysis of phthalates. Before taking the samples, all bottles were flushed with double-distilled water, then with high purity acetone free of the tested PAE residues. Influent was collected before mechanical treatment, and effluent wastewater was collected at the outlet to the Utrata River near the MWWTP. After delivery to the laboratory, all samples were filtered under pressure using a 1.2 μm glass filter (washed in acetone) and, subsequently, frozen at −20 °C until analysis.

The three species of plants, *Cyperus papyrus*, *Lysimachia nemorum* and *Euonymus europaeus,* were also collected from the MWWTP; only the green part was taken to confirm the uptake of PAEs by plants in CWs. The plants were double-washed and dried in the laboratory for 3 days (temperature ≥ 23 °C). After that, the samples were dried at 60 °C for 3 h in a heating oven (Pol-Eko Aparatura, Wodzisław Śląski, Poland). The dried plants were homogenized using a mechanical blender (Kenwood, Havant, UK) and frozen at −20 °C until analysis. The average water content in *Cyperus papyrus*, *Lysimachia nemorum* and *Euonymus europaeus*, determined based on the weight of the sample before and after desiccation, was 75.4%, 64.7% and 68.5%, respectively.

### 3.4. Development of the Analytical Method for Determining Target Compounds in Wastewater Samples

Solid-phase extraction (SPE) was used for the extraction of phthalates from wastewater samples. In order to optimize the extraction conditions, three different cartridges, Oasis HLB, 6 mL/300 mg, StrataX, 3 mL/200 mg and Strata C18-ec, 6 mL/500 mg, were tested. Each cartridge was preconditioned with 5 mL of ethyl acetate (EtOAc), 5 mL of methanol (MeOH) and 5 mL of distilled water adjusted to pH 3 (using 1 M HCl). Next, the spiked distilled water samples, with each analyte at a concentration of 4 µg × L^−1^ (250 mL adjusted to pH 3), were passed through a cartridge at a flow rate of ~4.5 mL × min^−1^ using a vacuum manifold. After the sample was loaded, the sorbent was washed with 10 mL of a mixture of MeOH:H_2_O (1:9, *v/v*) and subsequently air-dried under a vacuum for 60 min. The adsorbed analytes were eluted with 2 × 5 mL of EtOAc and evaporated to dryness. Finally, the samples were reconstituted in 0.1 mL of acetone and analyzed by the GC–MS(SIM) method described in detail in Section 3.6. The extraction of non-spiked samples was carried out for each experiment.

In order to evaluate the usefulness of the analytical method for determining target compounds in wastewater samples, raw and treated sewage samples containing trace levels of PAEs were spiked with a known amount of the target phthalates (250 ng × L^−1^, 500 ng × L^−1^ and 1000 ng × L^−1^) and subjected to extraction 24 h after spiking (each sample in three replicates). The extraction of non-spiked samples was also carried out for each experiment. The absolute recovery (AR) of analytes from both types of matrices was evaluated according to the procedure described in Caban et al. [42] using Equation (1):

AR = ((C − D)/A) × 100%(1)

where A is the peak area of the analyte recorded for the standard solution, C is the peak area of the analyte recorded for the sample spiked with the target compound before extraction and D is the peak area of the analyte recorded for the non-spiked sample (blank sample). AR was presented as a mean value.

### 3.5. Development of the Analytical Method for Determining Target Compounds in Plant Materials

Ultrasound-assisted extraction (UAE) combined with SPE for cleaning the plant extracts was used for the extraction of phthalates from plant materials. The UAE extraction was performed using an SB 4200 DTD ultrasonic bath with temperature and power control systems (Polsonic, Warsaw, Poland). One gram of non-spiked dry papyrus (*C. papyrus*) material was put into a beaker, as well as material spiked with each analyte at a concentration of 1000 ng × g^−1^ dry weight (1 ± 0.01 g d.w.) (each sample was prepared in three replicates), together with 20 mL of one of the solvents ethyl acetate (EtOAc), methanol (MeOH) and dichloromethane (DCM), tested as the extraction medium. Such prepared samples were extracted under the following conditions: extraction time 30 min, operating frequency 40,000 Hz, temperature 25 °C. After this, the extracts were separated from the plant materials and decanted through a filter filled with 1 ± 0.01 g of sodium sulfate. The samples were evaporated to dryness and dissolved in 10 mL of acetone. Next, water to a volume of 250 mL was added to each extract, and the obtained solution was subjected to a cleaning procedure using the SPE procedure described in Section 3.4 (Oasis HLB cartridge). Finally, the samples were reconstituted in 0.1 mL of acetone and analyzed by the GC–MS(SIM) method described in detail in Section 3.6. The extraction of the non-spiked sample was also carried out. For appropriate equilibration, the spiked plant samples were extracted after 24 h of their storage under controlled temperature in the darkness.

The AR and ME values of analytes from plant materials were calculated as described in Caban et al. [42].

### 3.6. Chromatographic Conditions of GC–MS Measurements

The plant and wastewater extracts were analyzed using the GCMS-QP 2010 SE Shimadzu System (Shimadzu, Kyoto, Japan) with an AOC-5000 autosampler. The carrier gas was helium (100 kPa). The separation of analytes was carried out using a Zebron ZB-5MSi fused-silica capillary column (30 m, 0.25 mm I.D., 0.25 µm film thickness, Phenomenex). Injections (1 μL) were performed in the splitless injector mode (60-s). The temperature of the injector was 280 °C. The oven temperature program was 50 °C for 1 min, from 50 °C to 310 °C at 10 °C × min^−1^, and finally, 5 min at 310 °C (total time of analysis 32 min). The transfer line was held at 280 °C. The MS analysis (electron impact ionization 70 eV, temperature of the ion source 200 °C) was carried out using the single ion monitoring (SIM) mode. The scan time was 0.3 s. The time windows were 12.95–14.35 min, 14.35–15.73 min, 18.63–21.34 min, 21.34–24.02 min, 24.02–25.54 min and 25.54–26.77 min. The parameters used for identifying analytes were retention time, quantitative ions and confirmation ions, shown in Table 7.

### 3.7. Validation of the Proposed Methods for Determining Target Compounds in wastewater and Plant Samples

The proposed methods were validated using working calibration standard solutions and matrix-matched calibration solutions according to the guidelines of the International Vocabulary of Metrology [40].

The matrix-matched calibration solutions for determining target PAEs in treated and untreated wastewater were prepared by spiking samples with eight different concentrations of the target PAEs ranging from 7.8 to 1000 ng × L^−1^.

The matrix-matched calibration solutions for determining target PAEs in plant tissues were prepared by spiking plant samples with eight different concentrations of the target PAEs ranging from 19.5 to 2500 ng × g^−1^ d.w.

For each concentration level, three samples were prepared. Non-spiked samples were also analyzed.

The validation parameters linearity, correlation coefficient (R^2^), intermediate precision measurement (expressed by RSD, n = 3), mean recovery (MR), method detection limit (MDL) and method quantification limit (MQL) were established according to the procedures and calculations fully described in our previous papers [41,42].

The matrix effect (ME) was determined by spiking the appropriate amount of influent and effluent wastewater (250 mL) at concentrations of 250, 500 and 1000 ng × L^−1^ of each target compound, following the SPE procedure, and they were analyzed (in three replicates) according to the proposed GC–MS method. The extraction of each non-spiked water sample was also carried out. The same procedure was applied for the determination of the ME parameter for the plant materials (625, 1250 and 2500 ng × g^−1^ d.w). ME values were calculated according to the paper by Caban et al. [42] and presented as a mean value.

### 3.8. Application of the Proposed Methods for the Determination of Target Compounds in Wastewater and Plants Collected from an MWWTP

The previously developed and validated SPE–GC–MS(SIM) method for the determination of phthalates in wastewater samples was used to assess the number of target compounds in untreated and treated wastewater in an MWWTP. Wastewater samples (each in three replicates) were extracted and analyzed three times.

Among the plant species used in the MWWTP, three species were selected to assess the uptake of phthalates: papyrus (*C. papyrus*), European spindle (*E. europaeus*), yellow pimpernel (*L. nemorum*). The determination of the target compounds in plant materials was performed using the UAE–SPE–GC–MS(SIM) method proposed in this study. As with the wastewater samples, the plant samples (each in three replicates) were extracted and analyzed three times.

### 3.9. Evaluation of the Effectiveness of Removing Phthalic acid Esters in an MWWTP

The elimination efficiency (EE%) of target PAEs from the wastewater stream in an MWWTP supported by CWs was established according to the procedure described in our previous paper [41] based on concentrations of phthalates in treated (C_treated_) and untreated (C_untreated_) sewage (Equation (2)):

EE% = (C_untreated_ − C_treated_)/(C_untreated_) × 100% (2)

This parameter enables the effectiveness of removing target PAEs in the studied MWWTP to be described.

## 4. Conclusions

In this study, the analysis of the possibility of using hydroponic cultivation for the removal of six phthalates: dimethyl phthalate (DMP), diethyl phthalate (DEP), di-*n*-butyl phthalate (DBP), benzyl butyl phthalate (BBP), di-*n*-octyl phthalate (DOP) and bis(2-ethylhexyl) phthalate (DEHP), in a working full-scale MWWTP is presented. Three new plants, *Cyperus papyrus* (papyrus), *Lysimachia nemorum* (yellow pimpernel) and *Euonymus europaeus* (European spindle), were investigated for this purpose. For the first time, the uptake of PAEs by plants in a working full-scale MWWTP, which combines the method of biological wastewater treatment with AS and CWs, was determined.

The established elimination efficiency (EE) of the target compounds from wastewater ranged from 0% (DOP) to 98% (DEP). The EE value for DMP was not determined due to its concentration in wastewater below the MDL values. Among the three tested plant species, four of the six target PAEs were determined in *C. papyrus* (papyrus), and five in *L. nemorum* (yellow pimpernel) and *E. europaeus* (European spindle). Considering the sum of the uptake of the target PAEs by the plants, the highest uptake of phthalates was determined for *E. europaeus* (8938 ng × g^−1^ dry weight), thus, this plant was found to be optimal for supporting conventional MWWTPs. In summary, the application of the MWWTP–CW system allows for a significant increase in the removal of DEP, DBP, BBP and DEHP from the wastewater stream.

## Figures and Tables

**Table 1:** Absolute recovery (mean ± SD, %) of the target PAEs from wastewater samples spiked with the target phthalates to the concentrations: 250 ng × L^−1^, 500 ng × L^−1^ and 1000 ng × L^−1^, using Oasis HLB (n = 3).

Phthalates	Raw Sewage	Treated Sewage
	Mean ± SD (%)	
**DMP**	126 ± 5	118 ± 6
**DEP**	109 ± 17	121 ± 6
**DBP**	120 ± 25	113 ± 11
**BBP**	189 ± 25	137 ± 9
**DOP**	115 ± 13	39 ± 2
**DEHP**	108 ± 14	36 ± 2

**Table 2:** Absolute recoveries (mean ± SD) of six analytes using different solvents during the UAE procedure (n = 3, conc. 1000 ng × g^−1^ d.w.).

Type of Solvents/Phthalates	MeOH	EtOAc	DCM
	Value of Absolute Recovery [% ± SD (%)]		
**DMP**	29 ± 8	29 ± 16	32 ± 4
**DEP**	39 ± 10	60 ± 16	43 ± 8
**DBP**	62 ± 7	102 ± 31	83 ± 6
**BBP**	61 ± 6	80 ± 21	80 ± 4
**DOP**	120 ± 23	<MDL	130 ± 31
**DEHP**	14 ± 3	69 ± 26	68 ± 3

MeOH—methanol; EtOAc—ethyl acetate; DCM—dichloromethane.

**Table 4:** Concentrations of target compounds in raw and treated sewage samples collected from the studied full-scale MWWTP supported by CWs, determined using the developed SPE–GC–MS(SIM) method (n = 3).

Phthalates	Concentration in Raw Sewage	Concentration in Treated Sewage
	(mean ± SD) [ng × L^−1^]	
**DMP**	<MDL	<MDL
**DEP**	10,097 ± 202	178 ± 0
**DBP**	6196 ± 805	397 ± 8
**BBP**	204 ± 2	89 ± 0
**DOP**	221 ± 7	264 ± 3
**DEHP**	136 ± 0	41 ± 0

**Table 5:** Results of the determined target compounds in three species of hydroponically cultivated plants from an MWWTP using the developed UAE–SPE–GC–MS(SIM) method (n = 3), and the elimination efficiency of these compounds from wastewater in an MWWTP supported by CWs.

Phthalates	*Cyperus papirus* (Papyrus)	*Lysimachia nemorum* (Yellow Pimpernel)	*Euonymus europaeus* (European Spindle)	EE
	(Mean ± SD) [ng × g^−1^ Dry Weight]			%
**DMP**	<MDL	98 ± 2	397 ± 12	- ^1^
**DEP**	400 ± 24	313 ± 38	477 ± 83	98
**DBP**	1596 ± 215	1697 ± 140	1284 ± 278	94
**BBP**	1913 ± 146	<MDL	218 ± 19	56
**DOP**	1828 ± 196	1343 ± 193	6562 ± 1065	0 ^2^
**DEHP**	<MDL	53 ± 22	<MDL	70

^1^ if phthalate concentrations were below the MDL value in both raw and treated wastewater, the elimination efficiency (EE) was not calculated. ^2^ for DOP, the concentration in treated wastewater was higher than in raw. Based on this, it was assumed that the elimination efficiency was 0% and another source of DOP contamination exists in the WWTP.

**Table 6:** The sum of the uptake of selected phthalates in ng × g^−1^ dry weight by tested plant species growing in an MWWTP.

Plant Species	*Cyperus papyrus*	*Lysimachia nemorum*	*Euonymus europaeus*
**∑_selected PAEs_**	[ng × g^−1^ dry weight]		
	5737	3504	8938

**Table 7:** Retention parameters (time allowed change ± 0.15 min), time windows and SIM ions for the target compounds (quantitative and confirmation ions; quantitative ions are marked in bold).

Number	Phthalates	Retention Time (Rt) [min]	Characteristic Ions (*m*/*z*) (Quantitative and Confirmation Ions)	Time Windows [min]
**1**	DMP	13.250	**163**; 135; 164	12.95–14.35
**2**	DEP	15.060	**149**; 150; 177	14.35–15.73
**3**	DBP	19.225	**149**; 205; 223	18.63–21.34
**4**	BBP	23.015	**149**; 123; 206	21.34–24.02
**5**	DOP	24.575	**149**; 150; 279	24.02–25.54
**6**	DEHP	26.035	**149**; 167; 261	25.54–26.77

## Data Availability

Not applicable.

## Supplementary Materials

The following are available online: Table S1. Literature data concerning on the studies of usefulness of hydroponically cultivated plants for removing target phthalic acid esters (PAEs) from sewage stream, Table S2. Literature data concerning on the determination of selected PAEs in plant materials, Table S3. Chemical structures and physicochemical properties of selected phthalic acid esters (phthalates), Table S4. Main technological parameters of the studied MWWTP (average values from 2017), Figure S1. Mass spectra of target compounds with the MS fragments assignation, Figure S2. Activated sludge chamber with a system of constructed wetlands in the investi-gated Municipal Wastewater Treatment Plant in Sochaczew (Mazowieckie Voi-vodeship, Poland), Figure S3. Examples of total ion chromatograms (TICs) recorded for raw (A) and treated (B) wastewater samples, Figure S4. Example chromatogram with marked SIM ions for determined target compounds in real Papyrus (*Cyperus papyrus*) samples, Figure S5. Example chromatogram with marked SIM ions for determined target compounds in real Yellow pimpernel (*Lysimachia nemorum*) samples, Figure S6. Example chromatogram with marked SIM ions for determined target compounds in real European spindle (*Euonymus europaeus*) samples.
