# Supplementary material for: Evaluation of the Removal of Selected Phthalic Acid Esters (PAEs) in Municipal Wastewater Treatment Plants Supported by Constructed Wetlands
Source: Molecules. 2021 Nov 18;26(22):6966. doi: 10.3390/molecules26226966 (PMC8621385; doi:10.3390/molecules26226966)
Supplement: Supplementary file 1 [file molecules-26-06966-s001.zip › molecules-1422620-supplementary.pdf]

molecules

Supplementary Material

Article

Evaluation of the removal of selected phthalic acid esters (PAEs) in municipal wastewater treatment plants supported by constructed wetlands

Daniel Wolecki [1,*], Barbara Trella [1], Fei Qi[2], Piotr Stepnowski[1], Jolanta Kumirska [1,*]

[1] Department of Environmental Analysis, Faculty of Chemistry, University of Gdansk, Wita Stwosza 63, 80-308 Gdańsk, Poland; daniel.wolecki@phdstud.ug.edu.pl (D.W.); Barbara (B.T.); piotr.stepnowski@ug.edu.pl (P.S.); jolanta.kumirska@ug.edu.pl (J.K.)

[2] Key Lab for Source Control Technology of Water Pollution, College of Environmental Science and Engineering, Beijing Forestry University, Beijing, 100083, PR; qifei@bjfu.edu.cn China (F.Q)

* Correspondence: jolanta.kumirska@ug.edu.pl; +48 58 523 50 14 (J.K.) daniel.wolecki@phdstud.ug.edu.pl; +48 58 523 51 93 (D.W.)

Tables and Figures content:

Table S.1 Literature data concerning on the studies of usefulness of hydroponically cultivated plants for removing target phthalic acid esters (PAEs) from sewage stream

Table S.2 Literature data concerning on the determination of selected PAEs in plant materials

Table S.3 Chemical structures and physicochemical properties of selected phthalic acid esters (phthalates)

Table S.4. Main technological parameters of the studied MWWTP (average values from 2017)

Figure S.1 Mass spectra of target compounds with the MS fragments assignation

Figure S.2 Activated sludge chamber with a system of constructed wetlands in the investigated Municipal Wastewater Treatment Plant in Sochaczew (Mazowieckie Voivodeship, Poland)

Figure S.3 Examples of total ion chromatograms (TICs) recorded for raw (A) and treated (B) wastewater samples

Figure S.4 Example chromatogram with marked SIM ions for determined target compounds in real Papyrus (*Cyperus papyrus*) samples

Figure S.5 Example chromatogram with marked SIM ions for determined target compounds in real Yellow pimpernel (*Lysimachia nemorum*) samples

Figure S.6 Example chromatogram with marked SIM ions for determined target compounds in real European spindle (*Euonymus europaeus*) samples

Table S.1 Literature data concerning on the studies of usefulness of hydroponically cultivated plants for removing target phthalic acid esters (PAEs) from sewage stream

**PAEs**	**Type of CW / laboratory system / full-scale system in WWTP**	**Plant**	**Method of determining target compounds in plant material**			**MQL [µg/g d.w.]**	**Concentration in plant material [µg/g d.w.]**	**Concentration in untreated sewage [µg/L]**	**Concentration in treated sewage [ng/L]**	**Elimination efficiency from wastewater stream (EE%)**	**Ref.**
			**Extraction**	**Clean-up**	**Detection**						
DBP	Laboratory system	*Phragmites austrails*	UAE (no detail in paper)	Column separation with alumina and silica gel	GC-MS	1 µg/L (IDL)	Leaves<br>0.243 – 0.597 spring<br>0.482 – 1.610 summer<br>0.468 – 4.000 autumn<br>Details shown in Fig. 3 in paper	n.d.	n.d.	> 89.7<br>Details shown in Tab. 2 in paper	[16]
DBP	Laboratory system	*Phragmites austrails*	-	-	-	-	-	Experimental 500 (actual 80-410)<br>1000 (actual 280-860)<br>2000 (actual 480-1450)	Details shown in Fig. 2 in paper	62.08<br>82.17<br>84.17	[17]
DEP<br>DBP	Laboratory system	*Typha sp.*	UAE (20 mL, dichloromethane:	SPE (C18)	GC-MS	-	-	144 (Exp I)<br>150 (Exp II)<br>480 (Exp I)<br>510 (Exp II)		48.2-61.5<br>55.8-67.5<br>48.3-59.4<br>52.5-65.0	[18]

DEHP			methanol mixture,15 min)					1074 (Exp I)<br>1410 (Exp II)		41.4-57.5<br>52.0-63.9<br>Details in Fig. 3 in paper	
DMP<br>DEP<br>DnPP<br>DIBP<br>DBP<br>DIHP<br>DEHP<br>DINP	Laboratory system	*Wolffia arrhiza*	-	-	-	-	-	23.9 ±0.91<br>20.05 ± 1.12<br>15.16 ± 0.98<br>14.59 ± 1.21<br>51.1± 2.08<br>< LOD<br>61.35 ± 2.94<br>35.96 ± 3.60	< LOD<br>< LOD<br>< LOD<br>< LOD<br>12.57 ± 1.06<br>< LOD<br>25.15 ± 1.98<br>< LOD	> 95.5<br>>95.6<br>97.5<br>>96.4<br>87.2<br>-<br>97.7<br>>95.6	[19]
DMP<br>DEP<br>DBP<br>BBP<br>DOP<br>DEHP	Laboratory system	*Thalia dealbata; Arundo donax var. versicolor*	-	-	-	-	-	2.1 ± 0.4<br>5.0 ± 1.1<br>9.2 ± 1.5<br>4.1 ± 0.<br>6.1 ± 1.0<br>12.5 ± 2.2	n.d.	45-83<br>44-83<br>23-66<br>40-60<br>29-45<br>19-49	[20]
DBP<br>DOP<br>BBP	Laboratory system	*Brassica juncea; Helianthus annuus*	Soaking with a 40:80 mL acetone: hexane solution; 8 h	Filtered and washed with 20 mL of acetone and 80 mL of hexane. Then shaken with 200 mL of water and	GC-FID	200	Stalk/root/leaves<br>21000/20000/123900<br>4000/600/95400<br>3000/500/76500	170 ppm<br>100 ppm<br>80 ppm<br>(Figure 5 in paper)	~ 20 ppm<br>~ 20 ppm<br>~ 20 ppm<br>(Figure 5 in paper)	88<br>78<br>80	[21]

				reextracted with 60 mL of hexane							
DEHP<br><br>DiBP<br><br>DEP<br><br>DBP	Pilot-scale	*Juncus effusus*	-	-	-	-	-	Winter 4.42<br>Summer 8.49<br>Winter 0.44<br>Summer 0.51<br>Winter 1.82<br>Summer 2.71<br>Winter 0.38<br>Summer 0.16	n.d.	Winter 96<br>Summer 82<br>Winter ~95<br>Summer ~98<br>Winter ~73<br>Summer ~84<br>Winter ~76<br>Summer ~74	[22]
DBP	Pilot-scale	*Canna indica; Acorus calamus*	-	-	-	-	-	9840<br>19860	1.19<br>0.21	99.99<br>99.99	[23]
DEP<br><br>DBP<br><br>DEHP	Pilot-scale	*Phragmites australis*	-	-	-	-	-	0.151-3.788<br>(mean ~ 0.680)<br>0.043-6.134<br>(mean ~ 0.770)<br>0.040-2.480<br>(mean ~0.180)	~ 0.05 (HF)<br>~ 0.03 (VF)<br>~ 0.06 (HF)<br>~ 0.025 (VF)<br>~ 0.610 (HF)<br>~ 0.900 (VF)<br>Fig. 2 in paper	DEP and DBP highly removed DEHP an opposite trend	[24]
DBP	Pilot-scale	*Canna indica; Acorus calamus*	-	-	-	-	-	9840	5.82	99.84	[25]
Among 16 PAEs 10 compounds were found. DBP, DIBP, BEHP	Full-scale	*Phragmites austrails; Iris pseudacorus; Zizania caduciflora*	-	-	-	-	-	Cumulative concentration of PAEs in treated sewage and untreated waters for summer and winter season was presented. PAEs was increased by 60.26% in summer		Negative effect of the constructed wetland on PAEs was	[26]

and DEP were the dominant pollutants								and 26.43% in winter, respectively.		found in both summer and winter.	
15 PAEs including all investigated in this study	Full-scale an operational horizontal subsurface flow CW that receives effluent from an adjacent WWTP	The dominant plant species (*Typha latifolia, Typha angustifolia*)	UAE 1:1 v/v hexanes:acetone by tube rotation (10 min), sonication (15 min), and centrifugation (15 min at 3000 rpm)	SPE (Florisil 500 mg)	GC-MS	2.8-23.3 ppb (LOQ)	Sum phthalates 1.23 ± 0.53 µg g^{-1} for *Typha* shoots	n.d.	Sum phthalates 0.34 ± 0.13 µg L^{-1}	Results indicate reduction of phthalates in water exiting the CW. Significant concentrations of DBP, DEHP, and other water-soluble phthalates were found in the shoots of *Typha*, indicating plant uptake as a potentially important removal mechanism	[27]
DBP	Small-scale	*Canna indica; Acorus salamus*	Investigated enzymatic activity of DBP degradation only in soil during 30 days					Initial concentration 500 mg/g soil		On the 10th day, 35% in the surface	[28]

soil and 62% in the subsurface soil remained. After 30 days, 95.7% DBP in the surface soil, and 64.2% DBP in the subsurface soil.

Abbreviations: n.d. - not described in paper; dimethyl (DMP), diethyl (DEP), dipropyl (DnPP), dibutyl (DnBP), diisobutyl (DIBP), bis(2-ethylhexyl) (DEHP), diizoheptyl (DIHP) and diizononyl (DINP). (PAEs) (dimethyl phthalate (DMP), diethyl phthalate (DEP), di-n-butyl phthalate (DBP), butyl benzylphthalate (BBP), di-n-octyl phthalate (DOP) and bis (2-ethyl) hexylphthalate (DEHP))

References (position identical as in the manuscript)

16. Li, T.; Fan, Y.; Cun, D.; Song, X.; Dai, Y.; Wang, F.; Wu, C.; Liang, W. Treatment Performance and Microbial Response to Dibutyl Phthalate Contaminated Wastewater in Vertical Flow Constructed Wetland Mesocosms. *Chemosphere* **2020**, *246*, 125635, doi:10.1016/j.chemosphere.2019.125635.
17. Qi, X.; Li, T.; Wang, F.; Dai, Y.; Liang, W. Removal Efficiency and Enzymatic Mechanism of Dibutyl Phthalate (DBP) by Constructed Wetlands. *Environmental Science and Pollution Research* **2018**, *25*, 23009–23017, doi:10.1007/s11356-018-2384-5.
18. Witthayaphirom, C.; Chiemchaisri, C.; Chiemchaisri, W. Optimization of Reactive Media for Removing Organic Micro-Pollutants in Constructed Wetland Treating Municipal Landfill Leachate. *Environmental Science and Pollution Research* **2019**, doi:10.1007/s11356-019-06010-3.
19. Kotowska, U.; Karpinska, J.; Kapelewska, J.; Kowejsza, E.M.; Piotrowska-Niczyporuk, A.; Piekutin, J.; Kotowski, A. Removal of Phthalates and Other Contaminants from Municipal Wastewater during Cultivation of Wolffia Arrhiza. *Process Safety and Environmental Protection* **2018**, *120*, 268–277, doi:10.1016/j.psep.2018.09.019.
20. Xiaoyan, T.; Suyu, W.; Yang, Y.; Ran, T.; Yunv, D.; Dan, A.; Li, L. Removal of Six Phthalic Acid Esters (PAEs) from Domestic Sewage by Constructed Wetlands. *Chemical Engineering Journal* **2015**, *275*, 198–205, doi:10.1016/j.cej.2015.04.029.
21. Zavoda et al. UPTAKE,SELECTIVITY, AND INHIBITION OF HYDROPONIC TREATMENT OF CONTAMINANTS. *JOURNAL OF ENVIRONMENTAL ENGINEERING* **2001**, *127*, 502–508, doi:10.1146/annurev.pharmtox.42.091901.115838.

22. Reyes-Contreras, C.; Matamoros, V.; Ruiz, I.; Soto, M.; Bayona, J.M. Evaluation of PPCPs Removal in a Combined Anaerobic Digester-Constructed Wetland Pilot Plant Treating Urban Wastewater. *Chemosphere* **2011**, *84*, 1200–1207, doi:10.1016/j.chemosphere.2011.06.003.
23. Liang, W.; Deng, J.; Zhan, F.; Wu, Z. Effects of Constructed Wetland System on the Removal of Dibutyl Phthalate (DBP). *Microbiological Research* **2009**, *164*, 206–211, doi:10.1016/j.micres.2006.11.013.
24. Masi, F.; Conte, G.; Lepri, L.; Martellini, T.; del Bubba, M.; Florence, I. Endocrine Disrupting Chemicals (EDCs) and Pathogens Removal in an Hybrid CW System for a Tourist Facility Wastewater Treatment and Reuse. *Proceedings of the 9th IWA International Conference on Wetland Systems for Water Pollution Control* **2004**, 461–468.
25. Zhao, W.Y.; Wu, Z. bin; Zhou, Q.H.; Cheng, S.P.; Fu, G.P.; He, F. Removal of Dibutyl Phthalate by a Staged, Vertical-Flow Constructed Wetland. *Wetlands* **2004**, *24*, 202–206, doi:10.1672/0277-5212(2004)024[0202:RODPBA]2.0.CO;2.
26. Xu, S.; Zhou, S.; Xing, L.; Shi, P.; Shi, W.; Zhou, Q.; Pan, Y.; Song, M.-Y.; Li, A. Fate of Organic Micropollutants and Their Biological Effects in a Drinking Water Source Treated by a Field-Scale Constructed Wetland. *Science of The Total Environment* **2019**, *682*, 756–764, doi:10.1016/j.scitotenv.2019.05.151.
27. Diepenheim, G.; Gift, S.C.; Harb, C.; Wallace, M.; Layshock, J. Survey of Phthalate Mitigation and Distribution in Water, Sediment, and Typha in a Fully Operational Constructed Wetland: A Pilot Study. Bulletin of Environmental Contamination and Toxicology 2020, 105, doi:10.1007/s00128-020-02915-9.
28. Zhou, Q.H.; Wu, Z.B.; Cheng, S.P.; He, F.; Fu, G.P. Enzymatic Activities in Constructed Wetlands and Di-n-Butyl Phthalate (DBP) Biodegradation. *Soil Biology and Biochemistry* **2005**, *37*, 1454–1459, doi:10.1016/j.soilbio.2005.01.003.

Table S.2 Literature data concerning on the determination of selected PAEs in plant materials

PAEs	Experimental conditions / Concentration in hydroponic Solution /Days	Plant	Method of determining target compounds in plant material				Recovery [%]	MQL [μg/g d.w.]	Concentration in plant material [μg/g d.w.]	Ref.
			Sample preparation	Extraction	Clean-up	Detection				
DBP	16 seeds planted in soils/ DBP 0, 10, 20, and 40 mg/kg / plant samples were collected after 14 (seedling stage), 24 (jointing stage), and 40 (booting stage) days	*Triticum aestivum*	Plant samples were rinsed with deionized water, dried with filter paper, and separated into roots and leaves. The root and leaf tissues were soaked in liquid nitrogen and stored at 80 °C prior to analysis	UAE<br>0.2-g of plant sample, 20 mL of n-hexane and dichloromethane mixture (1:1, v/v) in an ultrasonic water bath (50/60 Hz) for 15 min; centrifugation at 3000 rpm for 30 min. The supernatant was decanted, and the residue was extracted one more time using 20 mL fresh solvent mixture.	A glass column filled with alumina (bottom layer) and anhydrous sodium sulfate (top layer) rinsed with dichloromethane (5 mL) rinsed several times; the combined eluates were concentrated to dryness at 50 °C, dissolved in acetonitrile (1.5 mL), filtered through a 0.22-mm membrane	HPLC-UV	n.p.	n.p.	Presented in Fig 2 in paper<br>The DBP content of wheat roots grown in fluvo-aquic soil under DBP stresses of 10, 20, and 40 mg/kg decreased by 56.8% (54.4%), 44.2% (68.1%), and 44.3% (61.4%), respectively; the corresponding decreases for cinnamon soil equaled 63.0% (67.9%), 62.6% (66.1%), and 54.2% (57.3%), respectively, and those for brown soil were 45.5% (58.8%), 47.1%	[2]

									(67.6%), and 46.4% (59.0%), respectively	
DBP	11 test plants / DBP 5 mg /L; 21 days	*Lactuca sativa L.* var. *longifolia*	The plant samples were rinsed three times with deionized water, then dried at 70◦C, ground into a mortar and sieved to <2 mm in size	1.00 g sample in 2 mL n-hexane shaken with a rotating shaker at 160 rpm for 1 h. Residual DBP was extracted with n-hexane three times and then the extracts were combined to await analysis	Not performed	GC-ECD	96.5%	0.08 µg/mL (MDL)	roots, 3.35 ± 0.42 stems 2.74 ± 0.38 leaves 4.35 ± 0.42	[29]
DEHP	nine vegetables planted in each greenhouse with 3 m length and 1 m width/ studied three types of plastic films with a thickness of 0.04 mm, made of polyvinyl chloride (PVC), polyethylene (PE), and a	potherb mustard (*Brassica juncea*), bok choy (*Brassica chinensis*), celery (*Apium graveolens*), spinach (*Spinacia oleracea*), cabbage (*Brassica oleracea*), leaf of tube	Vegetable samples were freeze-dried, ground, and sieved to less than 0.2 mm. Plastic film samples were cleaned with distilled water, dried, and cut to chips with 2 mm x 2 mm	Vegetable samples: 5-10 g; Soxhlet extractor (24 h, 200 mL acetone/DCM (1:1, v/v) at 75°C)<br><br>Plastic film: 2-5 g; Soxhlet extractor (24 h, 200 mL of acetone/dichloromethane (1:1,v/v) at 55°C)	Glass chromatography column (25 cm long, 1 cm i.d.), packed with 3 cm alumina plus 10 cm silica, followed by 2 cm anhydrous sodium sulfate. Dichloromethane (20 mL) for elution. The collected fraction reduced to 0.5	GC-MS	n.d.	n.d.	The DEHP contents of nine vegetables were 15.75 ±0.87 -33.96 ± 2.46 µg /g (dry weight) from the greenhouse with the PVC film and 11.21 ±1.67-27.34 ±0.70 µg /g (dry weight) from the greenhouse with the PVC-PE composite film. For vegetables from the greenhouse	[30]

molecules MDPI

	PVC-PE composite / different height Greenhouses (0.5 m; 1.0 m; 1.5 m) / different age greenhouses (0 month; 3 months / after 6 months) / four PVC films with thicknesses of 0.02, 0.04, 0.06, and 0.08 mm	(*Allium tuberosum*), lettuce (*Lactuca sativa*), garlic (*Allium sativum*) and edible amaranth (*Amaranthus mangostanus*)			mL under a gentle stream of nitrogen				with the PE film, there were only three vegetables in which DEHP was detected. The DEHP concentrations of four vegetables (*B. juncea*, *A. graveolens*, *L. sativa*, and *A. mangostanus*) were higher than the other five vegetables. More information in Tables 1-3 in this paper	
DBP DEHP	15 seeds placed moistened with 5 mL of one of the prepared DBP or DEHP solutions ((5, 10, and 20 mg/mL). Control seeds were cultured in distilled water (blank control) or 2% methanol (methanol	wheat seedlings	Seedlings rinsed with distilled water and dried on filter paper. Shoot and root separated.	500 mg of shoots or roots mixed with anhydrous sodium sulfate (Na_2SO_4) and a small amount of silica sand. UAE, 20 mL CH_2Cl_2,15 min.	A chromatographic column with aluminum oxide (1 g) and anhydrous Na_2SO_4 (2 g) rinsed with 10 mL CH_2Cl_2. 20 mL CH_2Cl_2 was added to the column when the digests arrived to the Na_2SO_4 layer,	HPLC-UV	n.d.	n.d.	DBP shoot from 11.37 to 59.26 µg/g; root from 19.21 to 79.23 µg/g depending on the applied DBP concentration and harvested time. DEHP DEHP shoot from 6.14 to 35.31 µg/g; root from 12.06 to 49.39 µg/g depending	[31]

	control) / Germination experiments terminated when the growing radicle in the control was longer than 20 mm				next 10 mL CH_2Cl_2. Filtration of the eluat by 0.22 µm membrane				on the applied DEHP concentration and harvested time. More details in Table 3 in this paper	
DMP DEP DBP BBP DEHP DOP	Agriculture cultivation / over 110 samples of soil (paddy soil) and plant material o an agricultural area about 250 m, east China	*Medicago sativa* as green manure; vegetable plots: carrot, soybean, cauliflower, radish and pak choi	1.00 g of plant sample placed in a clean glass centrifuge bottle, was mixed on a vortex mixer for 1 min and immersed in 30 mL of acetone:hexane (1:1 v/v) overnight. Before UAE centrifugation of the sample at 1,500 rpm for 5 min	UAE (30 mL acetone:hexane, 1:1 v/v, 30 min at 25°C). The three supernatants were all filtered into a round-bottom flask after another two extractions with 20 mL of acetone: hexane (1:1 v/v) for 15 min each. Evaporation to 1–2 mL (350 mbar, 40°C water bath, 80 rpm), addition of hexane (3–4 mL) to the remaining solvent and evaporation to a volume less than 1 mL but not to dryness	Column chromatographic separation using a glass column with 2 g of Na_2SO_4, 6 g of neutral Al_2O_3 and 12 g of neutral silica gel (from bottom to top). Pre-washing step: 15 mL of hexane and 15 mL of acetone:hexane (1:4 v/v) mixture. Sample loading and elution with 40 mL of acetone:hexane	GC-MS	n.d.	Instrument detection limits (IDLs) for 6 target compounds 0.11–0.35 µg/L	The concentrations of six target PAE pollutants ranged from 0.31–2.39 µg/g in soil to 1.81–5.77 µg/g in various plants (dry weight/DW), and their bioconcentration factors (BCFs) ranged from 5.8 to 17.9. More detail in Figure 4 in this paper	[32]

					(1:4 v/v). Evaporation to less than 1 mL of rsolution					
DBP<br>DEHP	Laboratory system - 1 kg sand (dry weight) spiked with PAEs to concentration 500 µg/kg (dry weight) for each congener. Plant cultivation 28 days / 16 h light/8 h dark cycle	lettuce *Lactuca sativa;* strawberry *Fragaria x ananassa; Daucus* carrot *carota Var. Sativa*	After 28 days plants were separated into roots and leaves, rinsed with deionized water, freeze-dried, and ground to a fine powder using a stainless-steel coffee grinder. The dried plant tissues were stored at −20°C until extraction	UAE 0.2-g aliquot of plant sample (15 mL of n-hexane and dichloromethane mixture (1:1, v/ v) in an ultrasonic water bath (50/60 Hz) for 15 min, followed by centrifugation at 3000 rpm for 30 min. The residue was re-extracted using 15 mL fresh solvent mixture	Column separation with Al_2O_3 and silica gel. After elution with 40 mL of acetone/hexane (1:4 v/v), the eluent was reduced to about 1 mL	GC-MS	75–110%	3.3<br>8.5	The mean PAE concentrations, based on dry plant mass, ranged from 128 to 2391 µg/kg for DBP and from 654 to 1371 µg/kg for DEHP in the leaves and roots of the three species. Show in paper in Figure 1. Bioconcentration factors (BCFs) ranging from 0.16 ± 0.01 to 4.78 ± 0.59	[33]

Abbreviations: n.d. - not described in paper; dimethyl phthalate (DMP), diethyl phthalate (DEP), butyl benzyl phthalate (BBP), di-n-butyl phthalate (DnBP), bis (2-ethylhexyl) phthalate (DEHP), di-n-octyl phthalate (DnOP)

2. Gao, M.; Dong, Y.; Zhang, Z.; Song, Z. Metabolism and Distribution of Dibutyl Phthalate in Wheat Grown on Different Soil Types. *Chemosphere* **2019**, *236*, 124293, doi:10.1016/j.chemosphere.2019.07.024.
29. Liao, C.-S.; Nishikawa, Y.; Shih, Y.-T. Characterization of Di-n-Butyl Phthalate Phytoremediation by Garden Lettuce (Lactuca Sativa L. Var. Longifolia) through Kinetics and Proteome Analysis. *Sustainability* **2019**, *11*, 1625, doi:10.3390/su11061625.
30. Fu, X.; Du, Q. Uptake of Di-(2-Ethylhexyl) Phthalate of Vegetables from Plastic Film Greenhouses. *Journal of Agricultural and Food Chemistry* **2011**, *59*, 11585–11588, doi:10.1021/jf203502e.

31. Gao, M.; Dong, Y.; Zhang, Z.; Song, W.; Qi, Y. Growth and Antioxidant Defense Responses of Wheat Seedlings to Di- n -Butyl Phthalate and Di (2-Ethylhexyl) Phthalate Stress. *Chemosphere* **2017**, *172*, 418–428, doi:10.1016/j.chemosphere.2017.01.034.
32. Ma, T.T.; Christie, P.; Luo, Y.M.; Teng, Y. Phthalate Esters Contamination in Soil and Plants on Agricultural Land near an Electronic Waste Recycling Site. *Environmental Geochemistry and Health* **2013**, *35*, 465–476, doi:10.1007/s10653-012-9508-5.
33. Sun, J.; Wu, X.; Gan, J. Uptake and Metabolism of Phthalate Esters by Edible Plants. *Environmental Science & Technology* **2015**, *49*, 8471–8478, doi:10.1021/acs.est.5b01233.

molecules

Table S.3 Chemical structures and physicochemical properties of selected phthalic acid esters (phthalates)

**Number**	**Trade name**	**pK_a/LogP**	**Water solubility [$mg\times L^{-1}$]**	**Chemical structure**
**1**	Dimethyl phthalate (DMP)	ND/1.6[b]	4[a] at 25 deg C	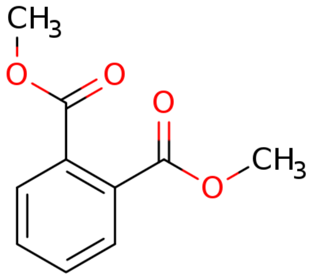
**2**	Diethyl phthalate (DEP)	ND/2.42[b]	1.080[a] at 25 deg C	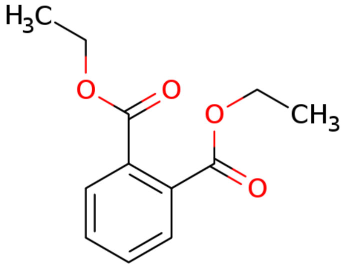
**3**	Di-n-butyl phthalate (DBP)	ND/4.5[b]	11.2[a] at 25 deg C	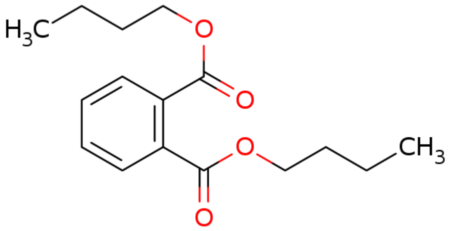
**4**	Benzyl butyl phthalate (BBP)	ND/4.73[b]	2.69[a] at 25 deg C	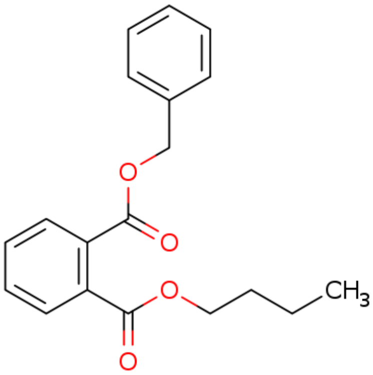

**5**	di-n-octyl phthalate (DOP)	ND/8.1[b]	0.022[c] at 25 deg C	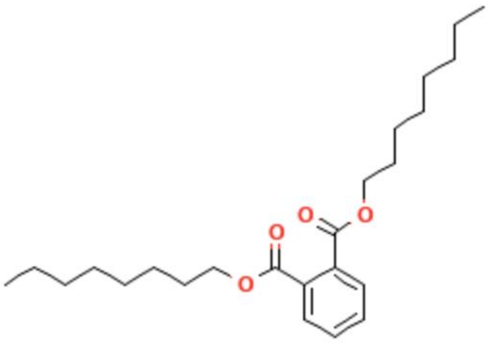
**6**	Bis(2-et-hylhexyl) phthalate (DEHP)	ND/7.6[d]	0.27[c] at 25 deg C	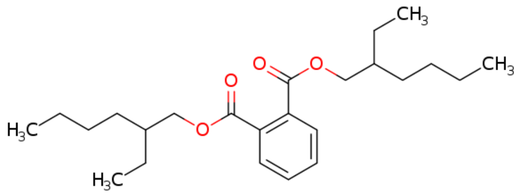

[a] based on Howard, P.H., Banerjee, S., Robillard, K.H., 1985. Measurement of water solubilities, octanol/water partition coefficients and vapor pressures of commercial phthalate esters. Environ. Toxicol. Chem. 4, 653–661. https://doi.org/10.1002/etc.5620040509

[b] based on EPA DSSTox (01.10.2019)

[c] based on U.S National Library of Medicine (01.10.2019)

[d] based on De Bruijn, J., Busser, F., Seinen, W., Hermens, J., 1989. Determination of octanol/water partition coefficients for hydrophobic organic chemicals with the "slow-stirring" method. Environ. Toxicol. Chem. 8, 499–512. https://doi.org/10.1002/etc.5620080607

Table S.4. Main technological parameters of the studied MWWTP (average values from 2017)

Factor	Results for untreated wastewater	Results for treated wastewater	The highest acceptable concentration	Removal efficiency
**BOD_5**	460 mg×L^{-1} O_2	3.4 mg×L^{-1} O_2	15 mg×L^{-1} O_2	99.3 %
**COD_{Cr}**	1144.7 mg×L^{-1} O_2	32.7 mg×L^{-1} O_2	125 mg×L^{-1} O_2	97.1 %
**N_{total}**	96.7 mg×L^{-1}	8.8 mg×L^{-1}	15 mg×L^{-1}	90.5 %
**P_{total}**	11.9 mg×L^{-1}	0.2 mg×L^{-1}	2 mg×L^{-1}	98.8 %
**Suspen-sions**	567.5 mg×L^{-1}	8.6 mg×L^{-1}	35 mg×L^{-1}	98.5 %
**pH**	7.9	7.2	-	-

BOD_5 Biochemical Oxygen Demand (for 5 days)

COD_{Cr} Chemical Oxygen Demand (using Potassium Dichromate)

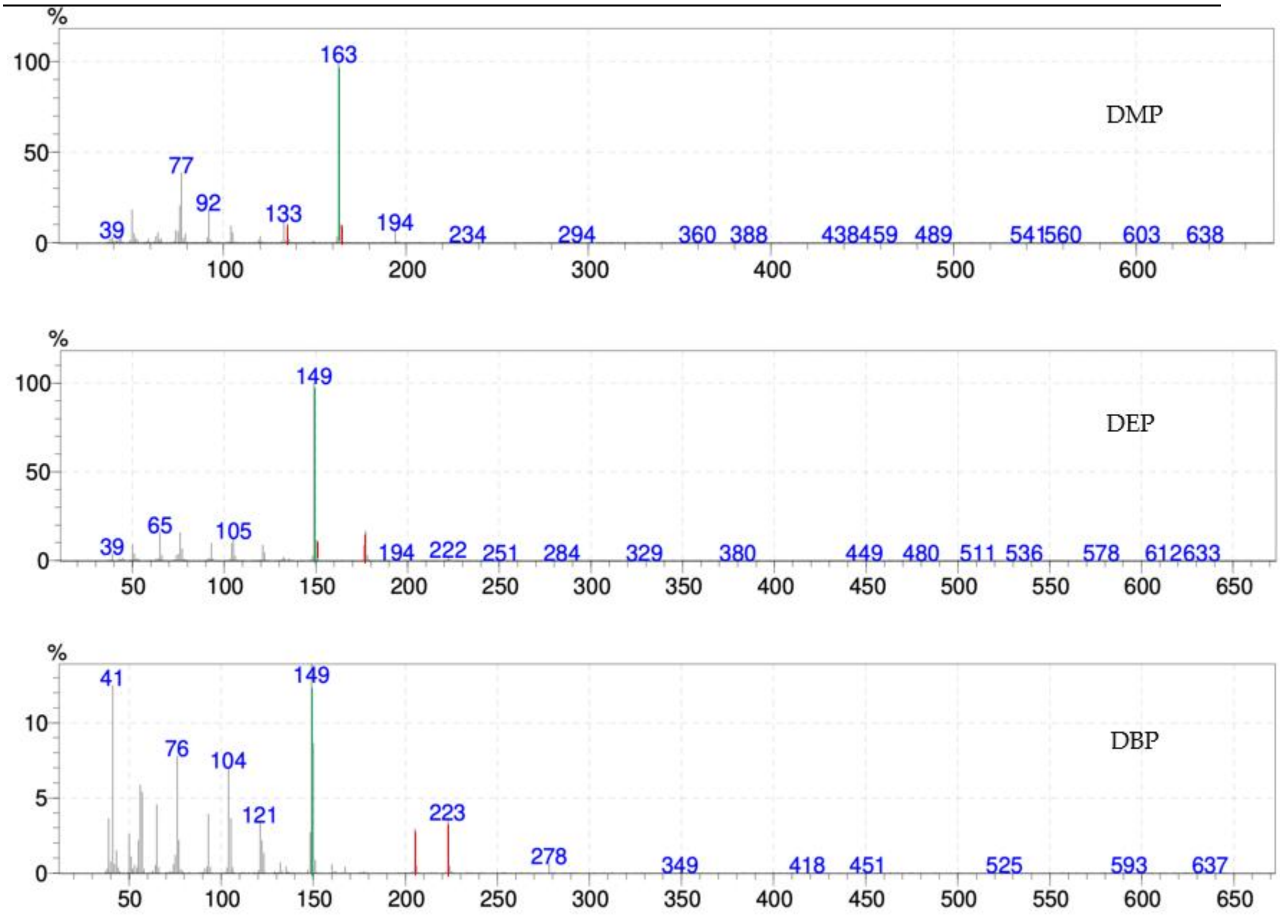

DMP

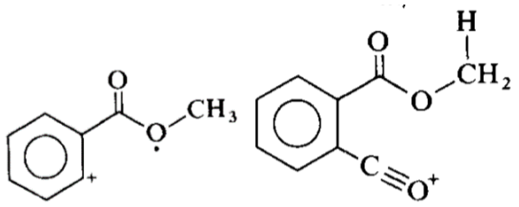

m/z 135 *m/z* 163

DEP

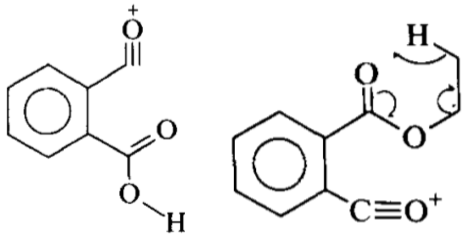

m/z 149 *n/z* 177

DBP

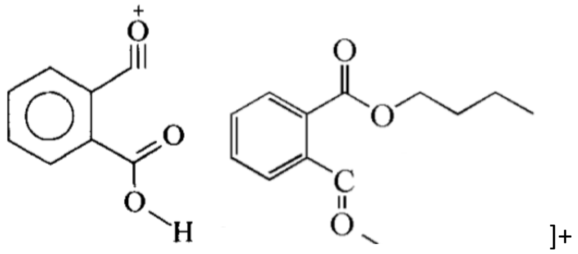

m/z 149 *m/z* 205

Figure S.1 Mass spectra of target compounds with the selected MS fragments assignation - part A

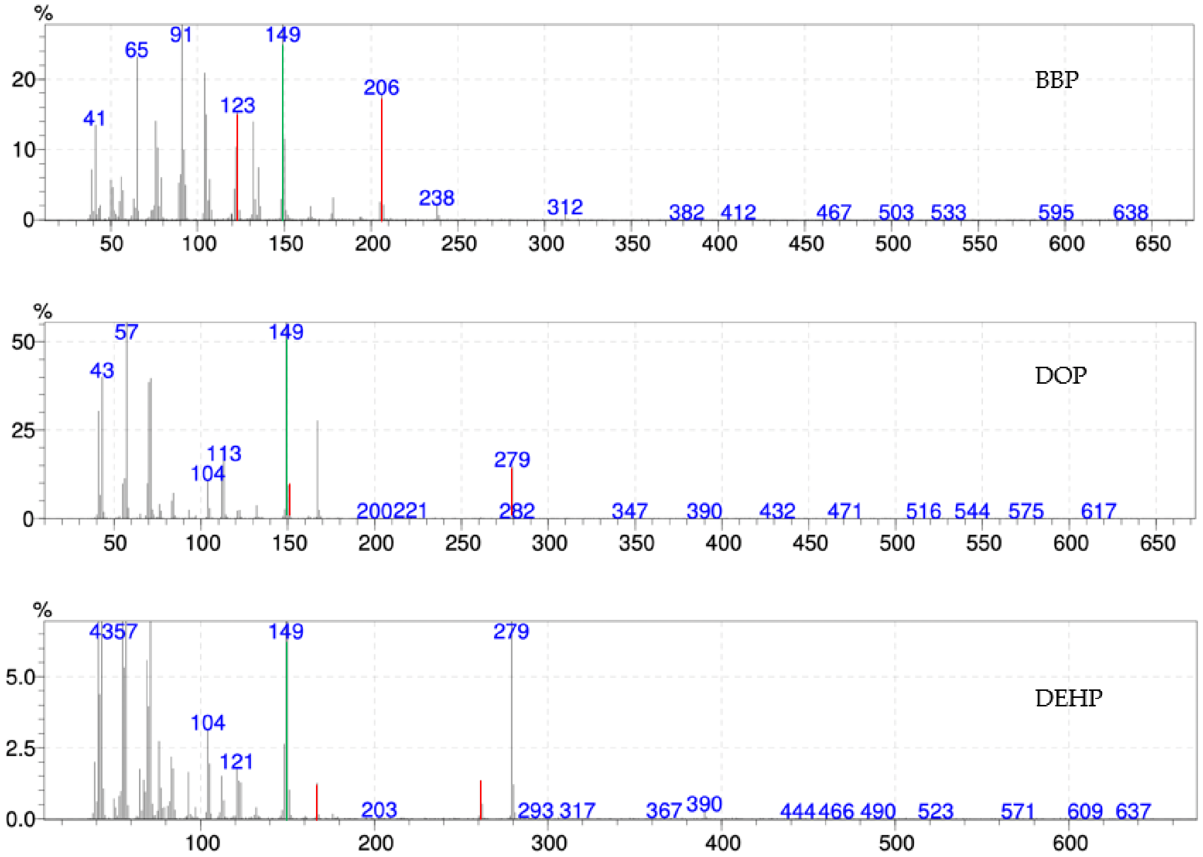

BBP

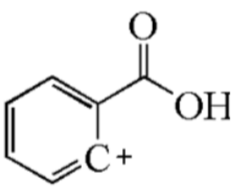

m/z 123

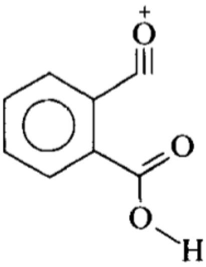

m/z 149

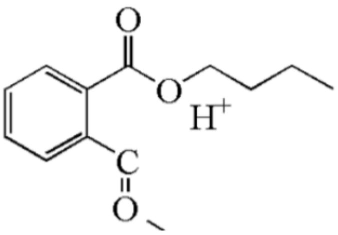

m/z 206

DOP

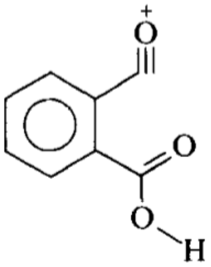

m/z 149

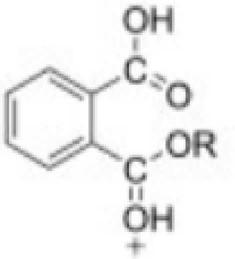

m/z 279

DEHP

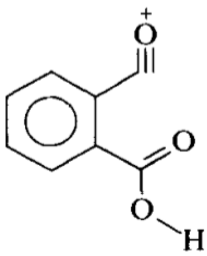

m/z 149

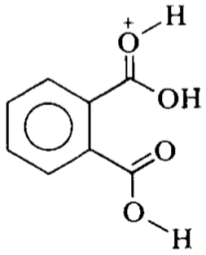

m/z 167

Figure S.1 Mass spectra of target compounds with the selected MS fragments assignation – part B

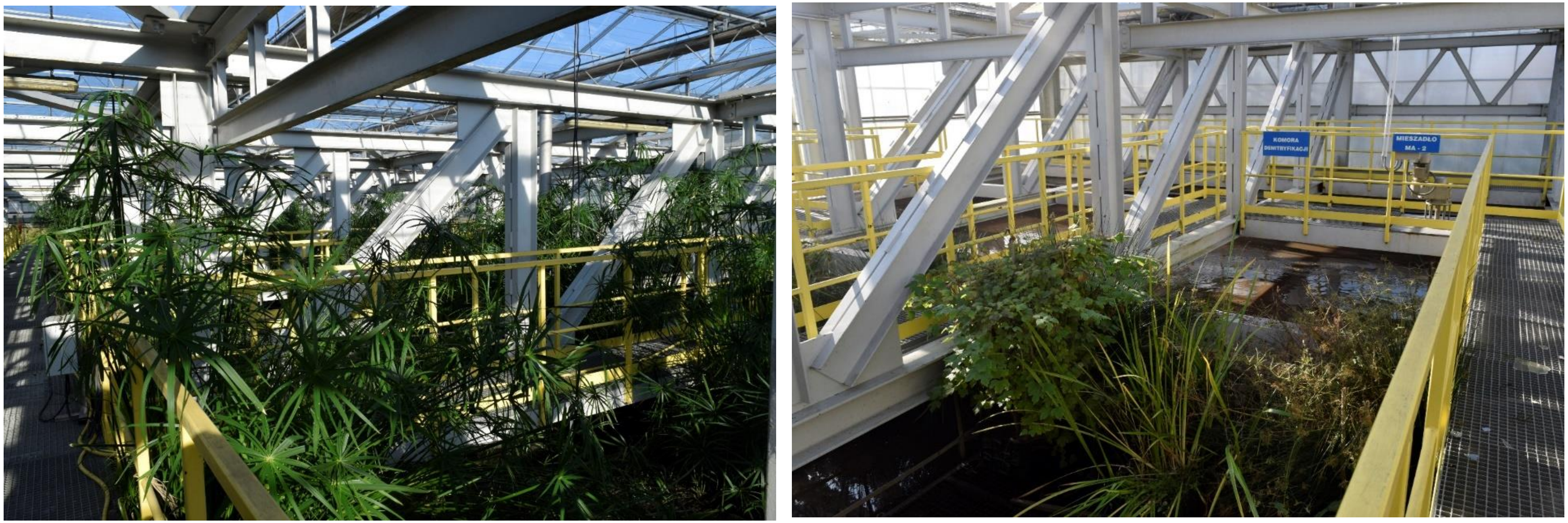

Figure S.2 Activated sludge chamber with a system of constructed wetlands in the investigated Municipal Wastewater Treatment Plant in Sochaczew (Mazowieckie Voivodeship, Poland

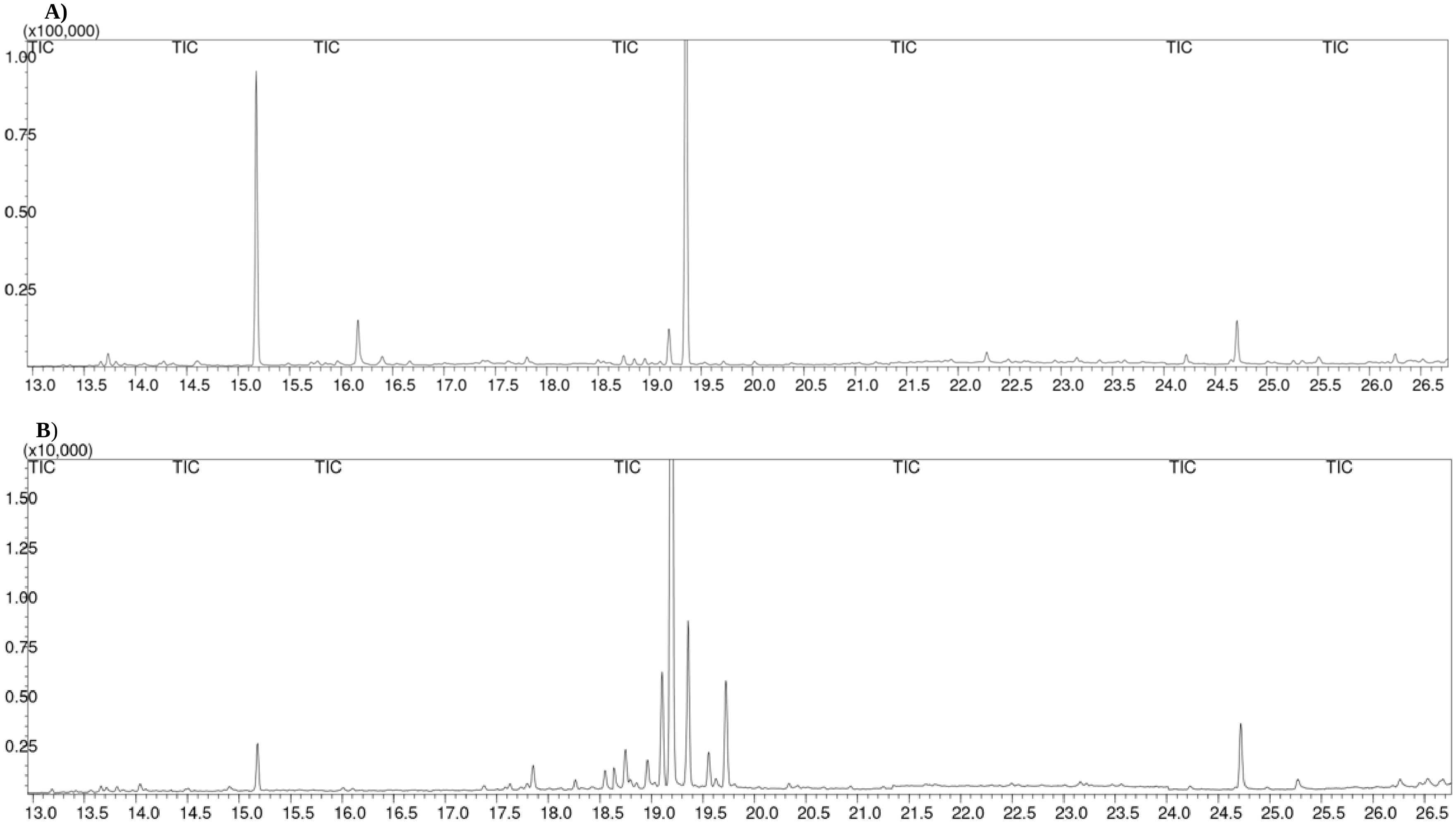

Figure S.3. Examples of total ion chromatograms (TICs) recorded for raw (A) and treated (B) wastewater samples

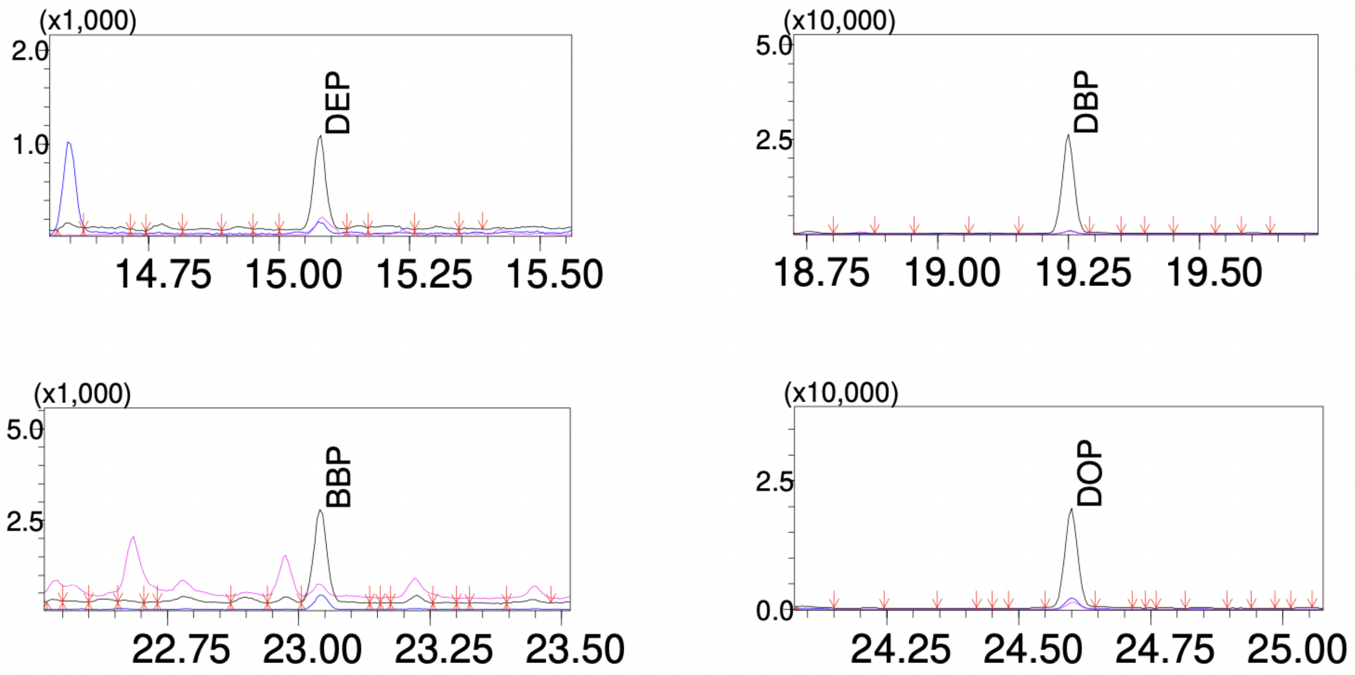

Figure S.4 Example chromatogram with marked SIM ions for determined target compounds in real Papyrus (*Cyperus papyrus)* samples.

molecules

MDPI

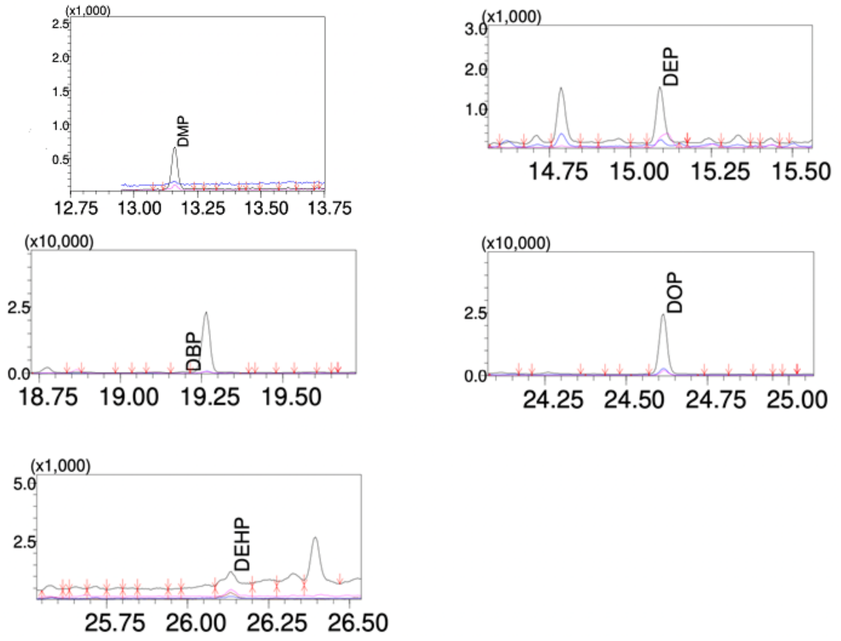

Figure S.5 Example chromatogram with marked SIM ions for determined target compounds in real Yellow pimpernel (*Lysimachia nemorum*) samples.

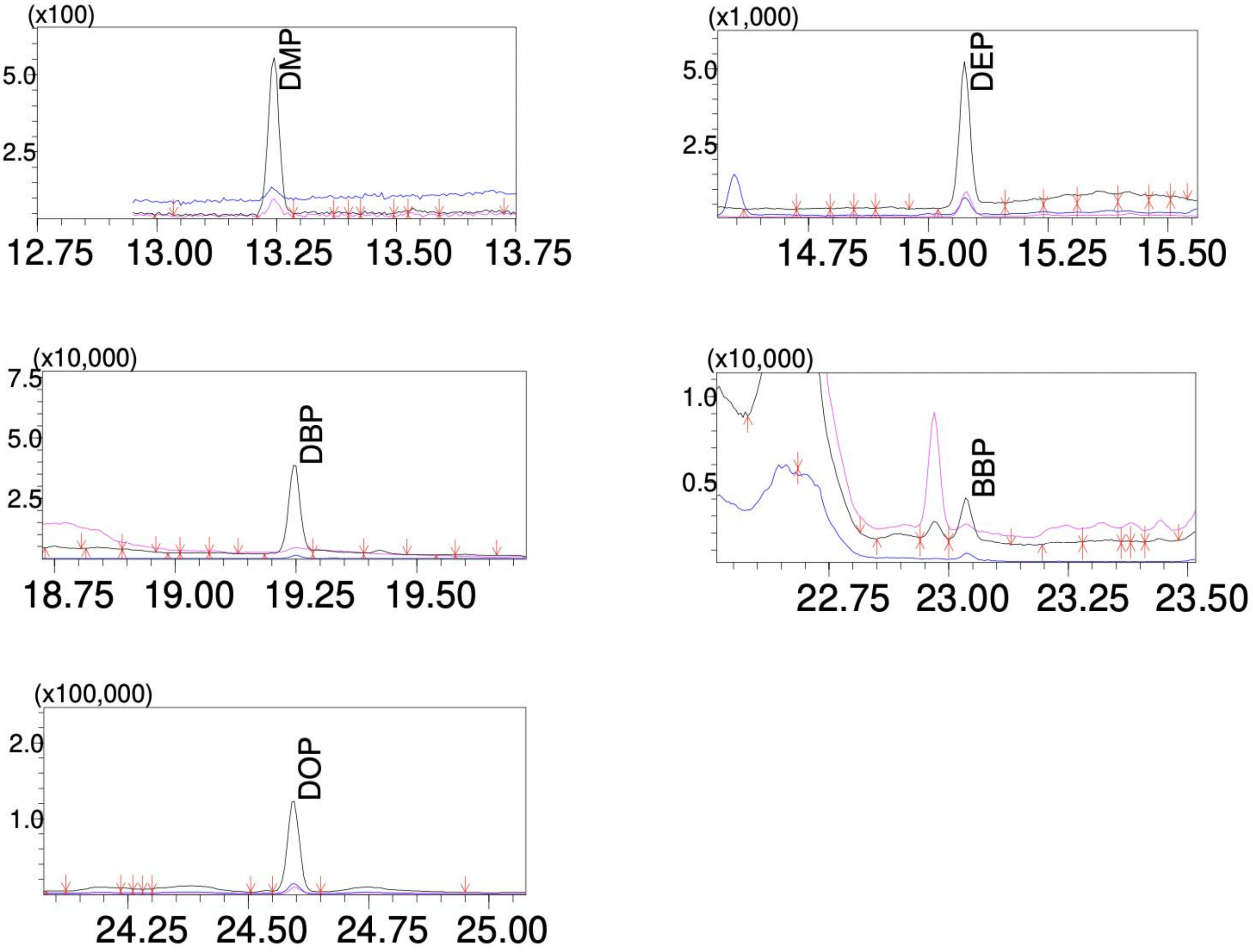

Figure S.6 Example chromatogram with marked SIM ions for determined target compounds in real European spindle (*Euonymus europaeus*) samples.
